# Assessing robustness of quantitative susceptibility-based MRI radiomic features in patients with multiple sclerosis

**DOI:** 10.1038/s41598-023-42914-4

**Published:** 2023-09-27

**Authors:** Cristiana Fiscone, Leonardo Rundo, Alessandra Lugaresi, David Neil Manners, Kieren Allinson, Elisa Baldin, Gianfranco Vornetti, Raffaele Lodi, Caterina Tonon, Claudia Testa, Mauro Castelli, Fulvio Zaccagna

**Affiliations:** 1 Department of Biomedical and Neuromotor Sciences, University of Bologna, Bologna, Italy; 2 Department of Information and Electrical Engineering and Applied Mathematics, University of Salerno, Fisciano, Italy; 3 UOSI Riabilitazione Sclerosi Multipla, IRCCS Istituto delle Scienze Neurologiche di Bologna, Bologna, Italy; 4 Department for Life Quality Sciences, University of Bologna, Bologna, Italy; 5 Functional and Molecular Neuroimaging Unit, IRCCS Istituto delle Scienze Neurologiche di Bologna, Bologna, Italy; 6 Department of Histopathology, Cambridge University Hospitals NHS Foundation Trust, Cambridge Biomedical Campus, Cambridge, United Kingdom; 7 Epidemiology and Statistics Unit, IRCCS Istituto delle Scienze Neurologiche di Bologna, Bologna, Italy; 8 Department of Physics and Astronomy, University of Bologna, Bologna, Italy; 9 NOVA Information Management School (NOVA IMS), Universidade NOVA de Lisboa, Campus de Campolide, 1070-312 Lisbon, Portugal; 10 Department of Imaging, Cambridge University Hospitals NHS Foundation Trust, Cambridge Biomedical Campus, Cambridge, United Kingdom; 11 Department of Radiology, University of Cambridge, Cambridge, United Kingdom; 12 Investigative Medicine Division, Radcliffe Department of Medicine, University of Oxford, Oxford, United Kingdom

**Keywords:** Multiple sclerosis, Image processing, Data processing, Biomarkers

## Abstract

Multiple Sclerosis (MS) is an autoimmune demyelinating disease characterised by changes in iron and myelin content. These biomarkers are detectable by Quantitative Susceptibility Mapping (QSM), an advanced Magnetic Resonance Imaging technique detecting magnetic properties. When analysed with radiomic techniques that exploit its intrinsic quantitative nature, QSM may furnish biomarkers to facilitate early diagnosis of MS and timely assessment of progression. In this work, we explore the robustness of QSM radiomic features by varying the number of grey levels (GLs) and echo times (TEs), in a sample of healthy controls and patients with MS. We analysed the white matter in total and within six clinically relevant tracts, including the cortico-spinal tract and the optic radiation. After optimising the number of GLs (n = 64), at least 65% of features were robust for each Volume of Interest (VOI), with no difference (*p* > .05) between left and right hemispheres. Different outcomes in feature robustness among the VOIs depend on their characteristics, such as volume and variance of susceptibility values. This study validated the processing pipeline for robustness analysis and established the reliability of QSM-based radiomics features against GLs and TEs. Our results provide important insights for future radiomics studies using QSM in clinical applications.

## Introduction

Multiple Sclerosis (MS) is an acquired autoimmune demyelinating disease affecting the Central Nervous System (CNS) and manifesting with a plethora of symptoms, including fatigue, limb sensory loss, paraesthesia, cognitive decline, and visual symptoms^1^. Currently, the diagnosis of MS is based on the McDonald diagnostic criteria, that combine clinical, imaging, and laboratory biomarkers^2^. Conventional Magnetic Resonance Imaging (MRI) has been a cornerstone in MS diagnostic criteria and in monitoring disease activity; however, standard techniques often demonstrate established lesions without providing information about the pathophysiological mechanisms leading to demyelination^3^.

Several quantitative MRI (qMRI) techniques, capable of in vivo quantification of imaging biomarkers, have been proposed to explore brain microstructure^4^ and metabolism^5^. In MS, these techniques have the potential to reveal pre-clinical inflammatory demyelination, affording a new therapeutic window. Quantitative Susceptibility Mapping (QSM)^6, 7^ is a qMRI technique sensitive to differences between the magnetic responses of adjacent tissues, returning the magnetic susceptibility voxel-by-voxel. A comparison between histological measurements and MRI-derived quantification of diamagnetic myelin and paramagnetic iron^8^ has confirmed that QSM can estimate the concentration of those substances in vivo and might be a suitable technique for providing *non-invasive* quantitative imaging biomarkers.

In patients with MS, chronic active or *smoldering* lesions are deemed to represent an ongoing subclinical inflammatory process, and present increased magnetic susceptibility at the edges due to the presence of iron-laden microglia and macrophages^9^, detectable by QSM. Interestingly, an increase in susceptibility within the basal ganglia and a decrease of thalamic susceptibility have been associated with higher disability^10^.

Over the past two decades, radiomics has emerged as a quantitative analytical tool for personalised medicine using medical imaging^11, 12^. Radiomics includes a collection of techniques that extract high-dimensional features from radiological images, most commonly using a Region/Volume of Interest (ROI/VOI) approach, including shape descriptors, intensity histogram and texture^13^. Since its inception, radiomics has been used to explore a wide array of medical image modalities, such as computed tomography^14^, MRI^15^, positron emission tomography PET^16^, hybrid imaging^17^, and photoacoustic imaging^18^. Clinical applications have also been broad including cancer imaging^19, 20^, chronic progressive illnesses^21^, and vascular disorders^22^. In neuroimaging, most attention has been focused on brain tumours using anatomical T_1_-weighted (T_1_w) and T_2_-weighted (T_2_w) images for tumour characterisation and grading^23, 24^, or assessing treatment response and clinical outcomes^25^.

Quantitative biomarkers able to characterise tissue features can be invaluable in providing information on disease state^26^. However, whenever an informative and relevant candidate biomarker is identified, its generalizability and replicability need to be confirmed. In fact, the radiomic pipeline from image acquisition to feature extraction is complex, with several parameters potentially influencing the reliability of results. To achieve clinical translation, robustness analysis of quantitative radiomic features is required^27–30^. The values of a robust feature do not strongly depend on the exact parameters and conditions used in the calculations.

QSM is intrinsically quantitative and lends itself naturally radiomic applications. However, a radiomics approach has rarely been applied to susceptibility-based imaging^31^ and, to our knowledge, no reports have been published to date on the reliability of QSM-derived radiomic biomarkers. This work aims to extract QSM-based radiomics features from Normal Appearing White Matter (NAWM) and clinically relevant WM tracts in healthy controls and patients with MS, and perform a robustness analysis to suggest potential features for use in classification, characterization, and prognosis, in patients with MS. The reliability of susceptibility features was assessed against the number of grey levels (GLs) and echo times (TEs); all details of the implemented pipeline are described in the following section.

## Materials and methods

### Study sample and MR examinations

We analysed the MR scans of 121 patients with MS (71F/50 M, 48.7 ± 12.6 years old [23–75]) and 30 Healthy Controls (HC) (17F/13 M; 53.4 ± 17.9 years old [24–86]), totalling 151 exams acquired at the IRCCS Istituto delle Scienze Neurologiche di Bologna (IT) (Bellaria hospital) between February 2020 and September 2022; the sample included multiple MS clinical phenotypes (relapsing remitting, primary and secondary progressive), to avoid phenotype-biased results. Acquisition and processing software and pipeline did not change over the course of the study, guaranteeing sample homogeneity and completeness. The size of the sample that we analysed is consistent with previous studies exploring susceptibility-based radiomics features, for example 140 subjects in Xiao et al*.*^32^, 172 in Zhang et al*.*^33^ and 149 in Kang et al*.*^34^.

The study was approved by the “Area Vasta Emilia Centro” Ethics Committee (CE-AVEC) (approval number AUSLBO 2023/CE 23043), and written informed consent was obtained from all participants. All methods were carried out in accordance with relevant guidelines and regulations.

All the scans were performed on a 3-T clinical scanner (Magnetom Skyra; Siemens Healthineers, Erlangen, Germany), using a 64-channel Head/Neck Coil as the receiver. The MR protocol included conventional morphological imaging (T_1_w Magnetization Prepared RApid Gradient Echo [MPRAGE] and T_2_w FLuid Attenuated Inversion Recovery [FLAIR]), Diffusion Weighted Imaging (DWI) and QSM. For QSM we employed 3D axial GRadient Echo (GRE), T_2_*w, 5 TEs, TE1/ΔTE = 53/9.42/9.42 ms, Time of Repetition (TR) = 53 ms, spatial resolution = 0.5 × 0.5x1.5 mm^3^, Flip Angle (FA) = 15°, acquisition time ~ 9′. The complete acquisition protocol is provided in Supplementary Materials (Supplementary Table S1). Quality control was performed by a single operator (with 5 years of experience in QSM imaging); no scans presented significant movement artifacts and all were considered suitable for analysis.

### Pre-processing pipeline and VOI segmentation

To obtain susceptibility images, phase maps from GRE T_2_*w measurements were processed, using Laplacian unwrapping^35^ and background field removal (Variable kernel Sophisticated Harmonic Artifact Reduction for Phase data^36^ [V-SHARP]) for each echo time; the five images obtained were combined via weighted averaging^37^ and dipole inversion was performed using the iterative least square (iLSQR^38^) method (STI Suite^39^). Cerebrospinal fluid was selected as a reference.

DWI images were skull-stripped (Brain Extraction Tool^40^ [BET]) from the Functional Magnetic Resonance Imaging of the Brain [FMRIB] Software Library^41^ (FSL) and denoised (dwidenoise function of MRtrix3^42^) using a principal component analysis approach; susceptibility-related distortion was estimated (topup function of FSL) and correction for susceptibility, eddy currents effects, and signal dropout was performed^43^ (eddy_openmp function of FSL). FLAIR, DWI, and QSM images were linearly registered (FSL’s Linear Image Registration Tool^44, 45^ [FLIRT]) to the corresponding MPRAGE images.

WM tissue was segmented using the MRtrix tool 5ttgen, based on FreeSurfer segmentation^46^. WM lesions were automatically segmented by the Lesion Prediction Algorithm^47^ as implemented in the Lesion Segmentation Tool (LST) version 3.0.0 (www.statistical-modelling.de/lst.html), an open-source toolbox for Statistical Parametric Mapping (SPM) (version 12); FLAIR images were used as input data^48, 49^. The toolbox provided an estimate for the lesion probability map, used to obtain a binary map of lesions: for each exam, the inverse of this map was multiplied by the WM mask to identify the NAWM. Even if LST provides a filling algorithm, we preferred not to use this option in our analysis after the identification of white matter lesions, to leave unchanged the intensity values of the original images as much as possible.

**Figure 1:**
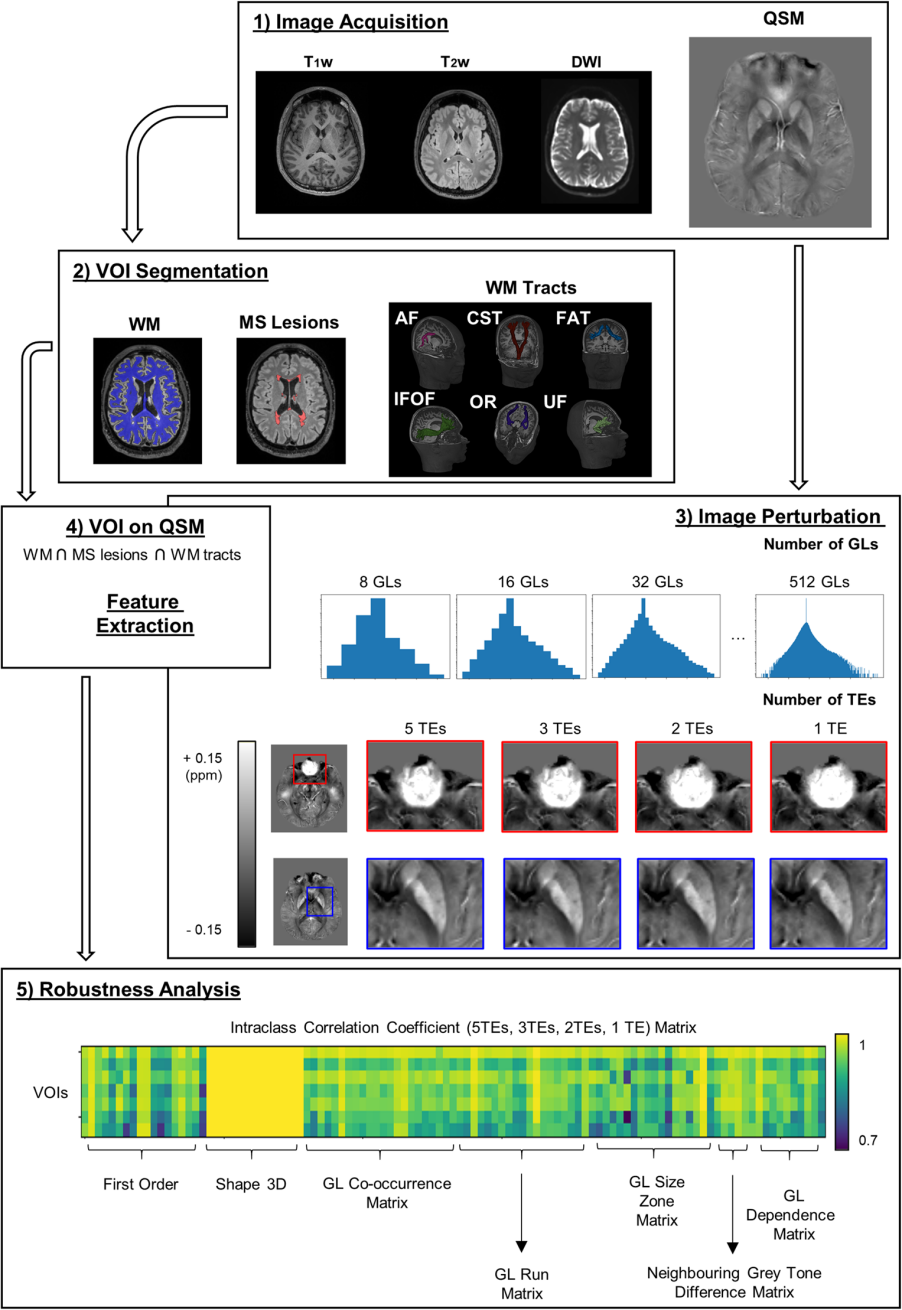
Scheme of the workflow. T_1_w MPRAGE, T_2_w FLAIR, DWI and QSM acquisition (M/25y, HC); (2) WM and MS lesions segmentation from T_2_w FLAIR (M/51y, MS) and WM tracts reconstruction from DWI (M/25y, HC); (3) image perturbation, changing the number of GLs and TEs (note changes in areas prone to artifacts (red boxes) and normal contrast (blue boxes) using different TEs) (4) VOIs overlap on QSM images and feature extraction; (5) Robustness analysis (Intraclass Correlation Coefficient). ICC was evaluated for each number of GL between QSM reconstructions with different TEs. ICC values for 3D shape features are equal to 1 for all the VOIs and all the measurements, because they depend only on the shape of the regions that we did not perturbate in this analysis; this class of features is not considered any further in this work. (T_1_w = T_1_-weighted, MPRAGE = Magnetization prepared RApid gradient echo, T_2_w = T_2_-weighted, FLAIR = fluid attenuated inversion recovery, DWI = diffusion-weighted imaging, QSM = quantitative susceptibility mapping, HC = healthy control, WM = white matter, MS = multiple sclerosis, GL = grey level, TE = echo time, VOI = volume of interest, ICC = intra-class correlation coefficient, AF = arcuate fasciculus, CST = cortico-spinal tract, FAT = frontal aslant tract, IFOF = inferior fronto-occipital fasciculus, OR = optic radiation, UF = uncinate fasciculus).

To reconstruct the WM tracts, diffusivity was modelled along the spatial eigenvector using the tensor model and high-order fibre modelling and probabilistic streamline approach used for crossing fibres evaluation^50^. The WM tracts analysed were: Arcuate fasciculus (AF), cortico-spinal tract (CST), frontal aslant tract (FAT), Inferior fronto-occipital fasciculus (IFOF), optic radiation (OR), Uncinate fasciculus (UF). A brief description of these structures is reported in the Supplementary Materials. The tractography pipeline was completely automatic, as were the other elements of the processing pipeline shown in Fig. 1. As NAWM, tract VOIs were analysed excluding the lesions.

### Radiomic feature extraction

As the data for the study were acquired using the same acquisition protocol, clinical scanner and processing pipeline at a single centre, the sample was deemed homogeneous rendering histogram normalization^25^ unnecessary. Pyradiomics^51^ 3.0.1 (Python 3.7.6) was used to extract features from 3D VOIs overlapped to QSM images; 107 features were extracted, divided into the following categories:

1. **First-order features (FO, # 18)** commonly used metrics to describe histogram intensity, including mean, median, 10th and 90th percentile, skewness, kurtosis; FO measurements are independent of the number of GLs
2. **Shape 3D features (S3D, # 14)** descriptors of the 3D size and shape of the VOI (e.g., volume, surface, minimum and maximum axes); S3D measurements are independent of the number of GLs and their intensity distributions
3. **Grey Level Co-occurrence Matrix features (GLCM, # 24)** describe the second-order joint probability function of an image region constrained by the mask
4. **Grey Level Run Length Matrix features (GLRLM, # 16)** quantify GL runs (number of consecutive pixels that have the same grey level value)
5. **Grey Level Size Zone Matrix features (GLZM, # 16)** quantify GL zones in an image (number of the connected voxels that share the same grey level intensity)
6. **Neighbouring Grey Tone Difference Matrix features (NGTDM, # 5)** quantify the difference between a GL value and the average grey value of its neighbours
7. **Grey Level Dependence Matrix features (GLDM, # 14)** quantify GL dependencies in an image (number of connected voxels within distance that are dependent on the central voxel)

Categories 3–7 are referred to as texture, and provide information about the spatial distribution of intensity levels in the image. A complete list of features can be found in the Supplementary Materials (Supplementary Tables S2 to S8).

### Robustness analysis of radiomic features

To assess the robustness of radiomic features, we evaluated:

- Numbers of GLs in the images, that affect texture features, and
- Numbers of TEs considered to produce the susceptibility maps. The number and values of TEs change with the acquisition system; their setting affects contrast and intensities in the resulting map. Normally, a compromise needs to be found between high (major contrast, both in well-reconstructed tissue and in areas with artifacts) and low values (that allow the visualisation of small details), and number of echo times (a larger number of echo times improves the Signal-to-Noise Ration [SNR] in multiple areas^52^, but increases acquisition time).

Seven different re-binning (2^n^, n ∈ [3, 4, …, 9] = 8, 16, 32, 64, 128, 256, 512 GLs) and four different QSM reconstructions were considered, using 5 (TE_i_, i ∈ [1, 2, 3, 4, 5]), 3 (TE_i_, i ∈ [1, 3, 5]), 2 (TE_i_, i ∈ [1, 5]) and 1 (TE_5_) echo times (TEs = 9.42, 18.84, 28.26, 37.68 and 47.10 ms). The Intraclass Correlation Coefficient (ICC)^53^ was measured to evaluate robustness, with 1 indicating perfect repeatability and 0 complete lack of resemblance. Following previous practice^54^, features above a threshold level of ICC > 0.85 were considered to have good reliability. Initial evaluation was performed both overall and by hemisphere (left *vs* right); the two-sample t-test was used to assess differences between the two sides, with significant *p-values* < 0.05 considered significant. The analysis was performed considering both controls and patients.

We estimated the optimal number of GLs and then evaluated changes in ICC between the different susceptibility maps. Shape 3D features are independent of intensity values. Considering that, to evaluate robustness we modified only the intensity distributions, ICC values did not vary across any binning configurations (Fig. 1). Hence, this class of features was not considered in the robustness analyses.

## Results

### Optimal number of GLs

**Figure 2:**
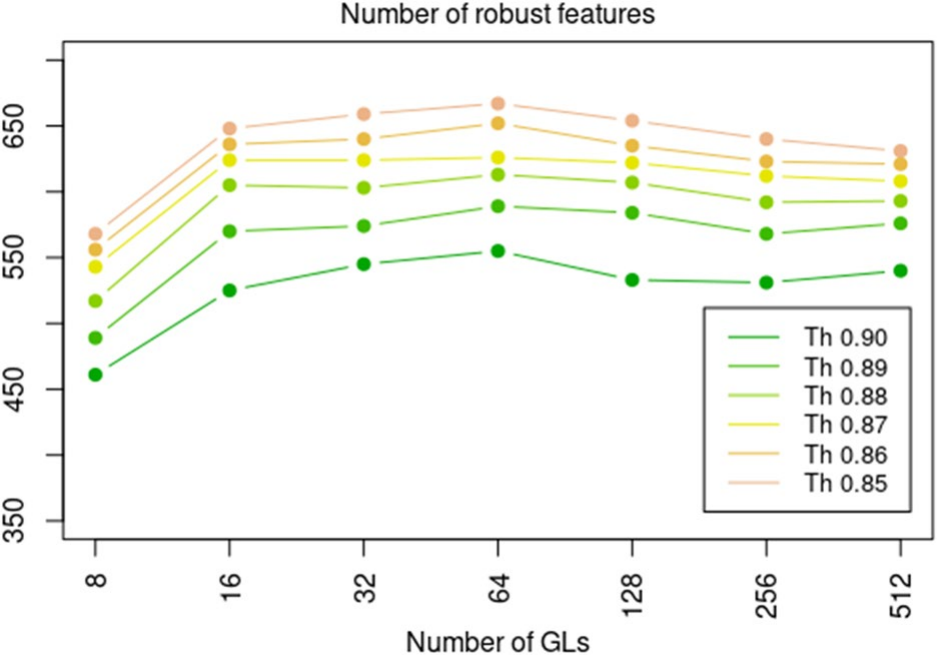
Optimal number of GLs. Line plot with number of robust features *vs* number of GLs used to quantize the images. Features were evaluated for all the VOIs (entire NAWM and 6 NAWM tracts), leading to a total number of 749 (= 7 VOIs × 107 features). A feature was considered robust when the ICC over the 4 (using different echo times) QSM reconstructions was higher than a set threshold level, from 0.85 to 0.90 (GL = grey levels, VOI = volume of interest, NAWM = normal appearing white matter, ICC = intraclass correlation coefficient, QSM = quantitative susceptibility mapping).

We evaluated the optimal number of GLs in QSM images to have the highest number of robust features. For each binning, from 8 to 512, ICC values between the 4 QSM reconstructions were evaluated in all the VOIs (NAWM Tracts: AF, CST, FAT, IFOF, OR, UF, and NAWM; 7 VOIs × 107 features = 749 features in total). Different threshold levels were explored at this stage, from 0.85 to 0.90; the trend in number of robust features and GLs for all the threshold levels is reported in Fig. 2. At all threshold levels tested, the maximum number of robust features was obtained using 64 GLs. Thus, we proceeded in our analysis using this binning. Numerical values are reported in the Supplementary Materials (Supplementary Table S9.). The same analysis was repeated extracting features by side (2 sides × 7 VOIs × 107 features = 1498) and considering patients and control groups independently. The results confirmed 64 GLs as the optimal binning.

### Robustness analysis

**Figure 3:**
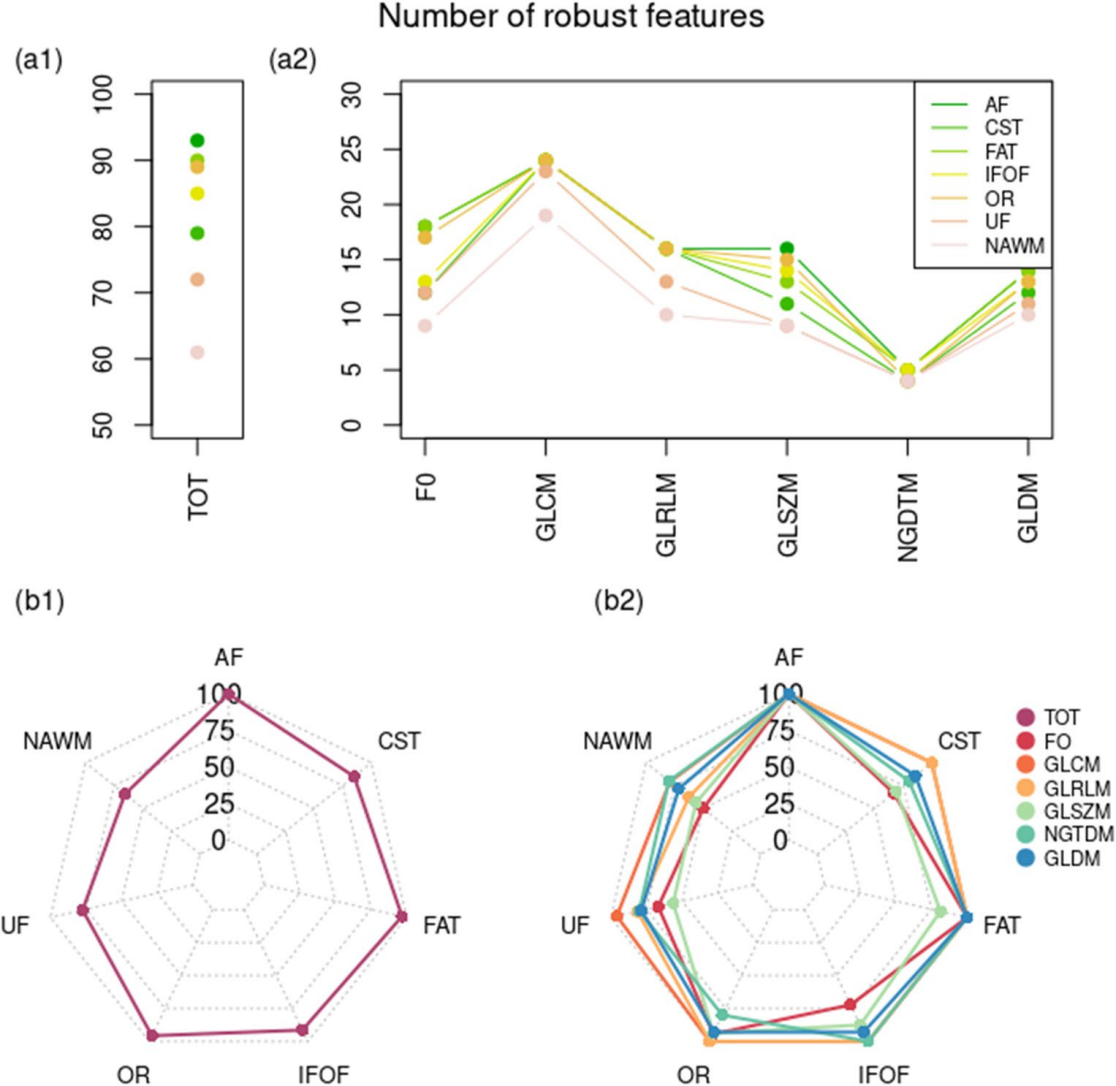
Robust features in NAWM and NAWM tracts. Features were extracted setting 64 as binning, and ICC was evaluated between the 4 (using different echo times) QSM reconstructions, setting 0.85 as threshold level. First row: line plot with number of robust features, total (a1) and considering the different feature categories (a2); second row: spider graph with the percentage of the number of robust features, total (b1) and considering the different feature categories (b2) (NAWM normal appearing white matter, ICC intraclass correlation coefficient, QSM quantitative susceptibility mapping, AF arcuate fasciculus, CST = cortico-spinal tract, FAT = frontal aslant tract, IFOF = inferior fronto-occipital fasciculus, OR = optic radiation, UF = uncinate fasciculus, FO = first-order, GL = grey level, GLCM = GL co-occurrence matrix, GLRLM = GL run length matrix, GLZM = GL zone matrix, NGTDM = neighbouring grey tone difference matrix, GLDM = GL dependence matrix).

In Fig. 3, robust features in NAWM and NAWM tracts are shown divided into different categories. The numerical values and the resulting ICC matrix are shown in the Supplementary Materials (Supplementary Figure S1 and Table S10). In NAWM, ~ 65% of features were robust; tracts were more robust with 75–100% robust features. There were differences in the different tracts: AF, FAT, and OR (~ 95–100% robust features) demonstrated more robust features than CST, IFOF and UF (~ 75–90%). Thus, we decided to explore the potential factors influencing those differences; the analysis is illustrated in the following subsection.

### First-order statistics analysis

**Figure 4:**
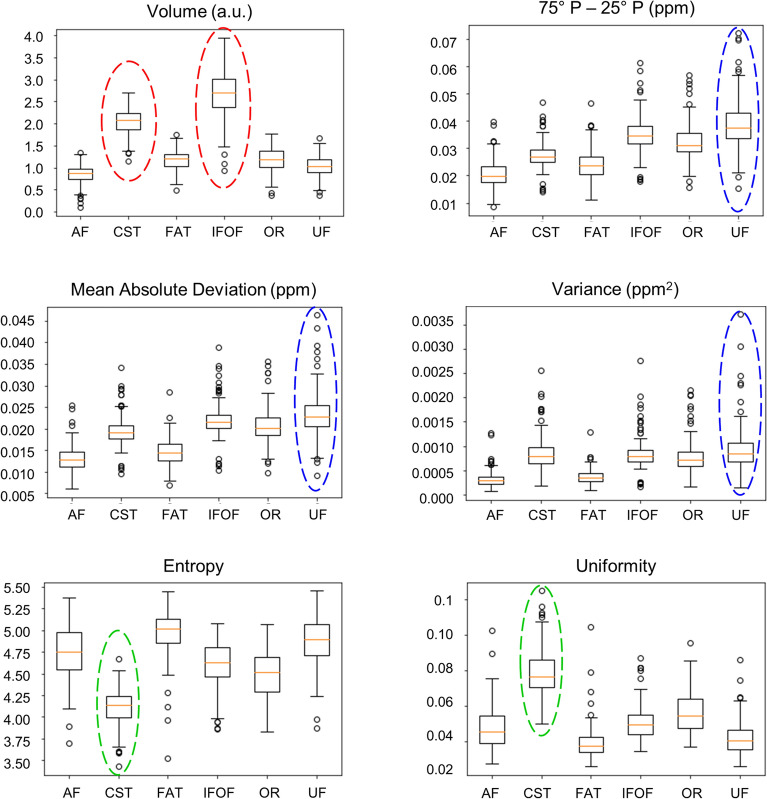
Volume and first order features in the NAWM tracts. Box plots of volume (corrected by the total brain volume according to the proportional adjustment method), interquartile range of susceptibility (= 75th P-25th P [P = percentile]), mean absolute deviation (MAD), variance, entropy and uniformity (first-order statistics features) distributions in the NAWM tracts (5TE-QSM). CST and IFOF have the greatest volumes (red dashed lines); UF has the highest variability in range, MAD, and variance (blue dashed lines); CST showed lower entropy and higher uniformity (green dashed lines) resulting in more uniform texture despite its volume (NAWM = normal appearing white matter, TE = echo time, QSM = quantitative susceptibility mapping, AF = arcuate fasciculus, CST = cortico-spinal tract, FAT = frontal aslant tract, IFOF = inferior fronto-occipital fasciculus, OR = optic radiation, UF = uncinate fasciculus).

Volume may play a significant role in robustness, indeed, NAWM, which has the largest volume, showed less robust features. Figure 4, top-left panel shows the volume distribution of the NAWM tracts: CST and IFOF, which showed fewer robust features compared to other tracts (e.g., AF and FAT), showed greater volumes, suggesting that the assumption that volume may possibly be influencing the number of robust features is maintained. Volumes of the structures of interest were corrected by using the proportional adjustment method^55^.

The UF also showed a lower number of robust features, but as opposed to CST and IFOF, its volume distribution was comparable with those of AF, FAT, and OR. Hence, we focused on the first-order features to further investigate the lower robustness of the UF.

ICC values for first-order features in the NAWM VOIs are reported in Table1 (ICC values for the other class of features are in the Supplementary Materials, Supplementary Tables S11 to S15). Review of individual values confirmed that, as mentioned in the previous section, AF, FAT, and OR showed higher robustness, while CST, IFOF, and UF, were more variable, similar to the overall NAWM. Most of the less-robust features in those VOIs (75th P-25th P, Mean Absolute Deviation, Range, Robust Mean Absolute Deviation, and Variance) are related to the degree of spread of the intensity histogram, which is generally higher in UF distributions (Fig. 4, ‘75°–25° P′, ‘Mean Absolute Deviation’ and ‘Variance’ panels).

**Table 1:** ICC values (n° GLs = 64) for the First-Order features in the analysed NAWM tracts and overall VOIs.

First-order features	NAWM tracts						NAWM
	AF	CST	FAT	IFOF	OR	UF	
10th P	**0.962**	**0.946**	**0.948**	**0.919**	**0.942**	**0.909**	**0.947**
90th P	**0.985**	**0.982**	**0.984**	**0.988**	**0.986**	**0.986**	**0.982**
Energy	**0.936**	**0.935**	**0.940**	**0.915**	**0.922**	**0.906**	**0.892**
Entropy	**0.976**	**0.908**	**0.968**	**0.928**	**0.952**	**0.888**	*0.839*
75th P-25th P	**0.906**	*0.830*	**0.899**	*0.810*	**0.883**	*0.815*	*0.834*
Kurtosis	**0.986**	**0.907**	**0.874**	**0.956**	**0.982**	**0.921**	*0.881*
Maximum	**0.944**	**0.916**	**0.962**	**0.903**	**0.914**	**0.852**	*0.729*
MAD	**0.893**	*0.842*	**0.887**	*0.783*	**0.870**	**0.791**	**0.811**
Mean	**0.979**	**0.973**	**0.973**	**0.969**	**0.974**	**0.961**	**0.983**
Median (50th P)	**0.979**	**0.972**	**0.972**	**0.970**	**0.973**	**0.960**	**0.982**
Minimum	**0.906**	*0.832*	**0.909**	**0.855**	**0.863**	*0.814*	*0.802*
Range	**0.893**	*0.833*	**0.914**	*0.833*	**0.853**	*0.780*	*0.717*
Robust MAD	**0.902**	*0.828*	**0.896**	*0.805*	**0.880**	*0.811*	*0.830*
RMS	**0.964**	**0.947**	**0.953**	**0.930**	**0.942**	**0.925**	**0.903**
Skewness	**0.988**	**0.943**	**0.978**	**0.943**	**0.981**	**0.958**	**0.956**
Total Energy	**0.936**	**0.935**	**0.940**	**0.915**	**0.922**	**0.906**	**0.892**
Uniformity	**0.979**	**0.919**	**0.969**	**0.936**	**0.956**	**0.893**	**0.845**
Variance	**0.872**	*0.848*	**0.860**	*0.724*	*0.843*	*0.741*	*0.732*

Considering 0.85 as the threshold, the robust features are highlighted in bold, the others in italic (ICC = intraclass correlation coefficient, GL = grey level, NAWM = normal appearing white matter, VOI = region of interest, P = percentile, MAD = mean absolute deviation, RMS = root mean squared, AF = arcuate fasciculus, CST = cortico-spinal tract, FAT = frontal aslant tract, IFOF = fronto-occipital fasciculus, OR = optic radiation, UF = uncinate fasciculus).

**Figure 5:**
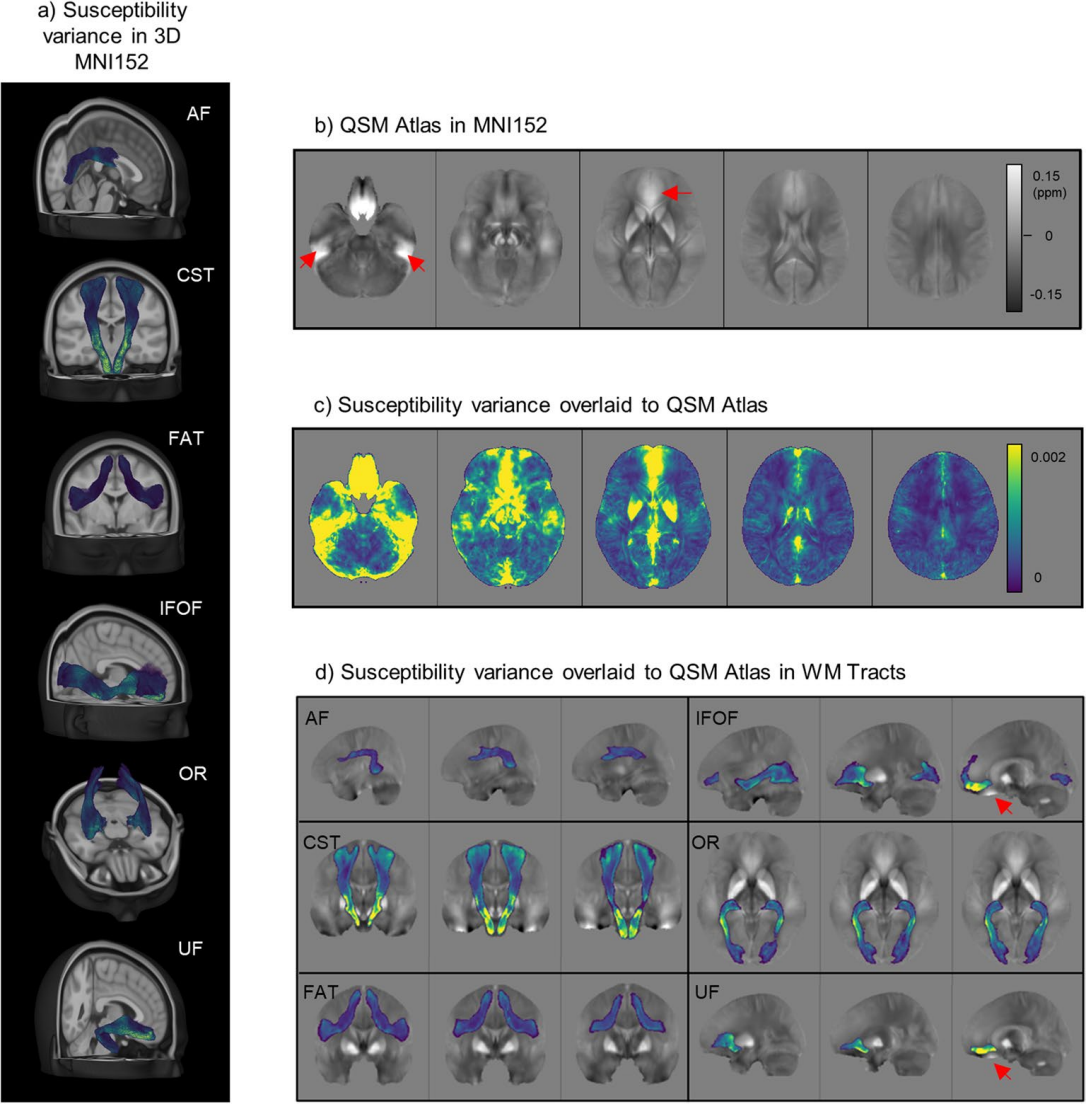
QSM atlas and susceptibility variance. QSM atlas was obtained non-linearly registering 30 HC exams to the MNI152 space; the variance of susceptibility measurements was evaluated on a voxel-by-voxel fashion—(**a**) susceptibility variance map in the 3D MNI152 in the white matter tracts; (**b**) QSM atlas in MNI152; (**c**) susceptibility variance map overlaid on QSM atlas; (**d**) susceptibility variance map reported individually for each WM tract, overlaid on QSM atlas, with the most representative projection for each of them (sagittal for AF, IFOF, and UF; coronal for CST and FAT; axial for OR). Red arrows in (**b**) and (**d**) indicate streaking artifacts on QSM reconstructions (QSM = quantitative susceptibility mapping, HC = healthy controls, MNI152 = Montreal Neurological Institute’s 152 space, WM = white matter, AF = arcuate fasciculus, CST = cortico-spinal tract, FAT = frontal aslant tract, IFOF = inferior fronto-occipital fasciculus, OR = optic radiation, UF = uncinate fasciculus).

Hence, we built a QSM-contrasted atlas in the Montreal Neurological Institute’s 152 space (MNI152) using QSM images of healthy controls to explore spatial relationships that could influence intensity values. In Fig. 5, the variance of the susceptibility values among the sample is shown voxel-by-voxel; the regions with more variability were areas prone to streak artifacts, i.e., closer to air-tissue boundaries or cortical bone, and the basal ganglia. Figure 5 also shows individual variance maps for each tract: CST, IFOF, and UF include areas with higher variance. In Supplementary Materials (Supplementary Figures S2 to S5) the CST susceptibility variance map is shown overlaid on the MNI152 space and on the QSM atlas.

### Texture analysis

Among first-order features, distributions of uniformity, which measures the homogeneity of the intensity values, and entropy, which measures the randomness in the image values, showed lower and higher median values in the CST compared to the other tracts (Fig. 4). Texture features (categories 3–7) allow for a more detailed description of the distribution of the intensity values: they measure fineness, coarseness and texture homogeneity and their trend shows that the CST is the one reporting less fine texture and more homogeneity in terms of texture, despite its wider histogram distribution, followed by IFOF and OR. In Table 2, a list of features supporting this observation. In Supplementary Materials (Supplementary Figures S6 and S7), the distributions of these features are shown.

**Table 2:** Second-order features measuring fineness, coarseness and texture homogeneity; their trend shows that the CST is the one reporting less fine texture and more homogeneity in terms of texture, despite its wider histogram distribution.

Texture category	Feature	Trend
GLCM	Difference Entropy	↓ CST
	Maximal Correlation Coefficient	↓ CST
	Joint Entropy	↓ CST
	Joint Energy	↑ CST
GLRLM	Short Run Emphasis	↓ CST
	Long Run Emphasis (*)	↑ CST
	Run Percentage (**)	↑ CST
	Gray Level Variance (***)	↓ CST
	Run Entropy	↓ CST, OR
	Run Length Non-Uniformity Normalised (****)	↓ CST
GLSZM	Long Area Emphasis (*)	↑ CST
	Zone Percentage (**)	↑ CST
	Gray Level Variance (***)	↓ CST, IFOF, OR
	Zone Entropy	↓ CST, OR
NGTDM	Complexity	↓ CST, IFOF
GLDM	Small Dependence Emphasis	↓ CST
	Large Dependence Emphasis (*)	↑ CST
	Dependence Non-Uniformity Normalised (****)	↓ CST
	Gray Level Variance (***)	↓ CST

CST = cortico-spinal tract, IFOF = inferior fronto-occipital fasciculus, OR = optic radiation, GL = grey levels, GLCM = GL co-occurrence matrix, GLRLM = GL run length matrix, GLSZM = GL size zone matrix, NGDTM = neighbouring grey difference tone matrix, GLDM = GL dependence matrix.

*Marks corresponding features in different categories.

GLRLM, GLSZM and GLDM describe the distribution of voxels measuring similar properties of different matrices, evaluating respectively length runs, size zones and dependencies. There are correspondences between the three classes with respect to the texture homogeneity of the CST: long run emphasis, long area emphasis and large dependence emphasis all show higher values for the CST; run and zone percentage both show higher values for the CST; GL variance, measuring the variance in GL intensity respectively for the runs, the sizes and the dependences, is lower in the CST distributions for the three categories.

CST has a larger volume compared to the other white matter tracts assessed in this study, hence, the variance of the intensity values is higher as expected; GL Non-Uniformity, which measures the similarity in intensity values, is higher for CST and IFOF for the three categories of GLRLM, GLSZM and GLDM (Supplementary Figure S8).

Other tracts, such as AF and FAT, which have more reliable features, show results in keeping with more fine texture: for example, Contrast in GLCM is higher in AF and FAT; Difference Entropy in GLCM, lower in CST, is higher in AF and FAT; Complexity in NGDTM, lower in CST and IFOF, is higher in FAT.

### Robustness analysis in NAWM tracts, left and right

**Figure 6:**
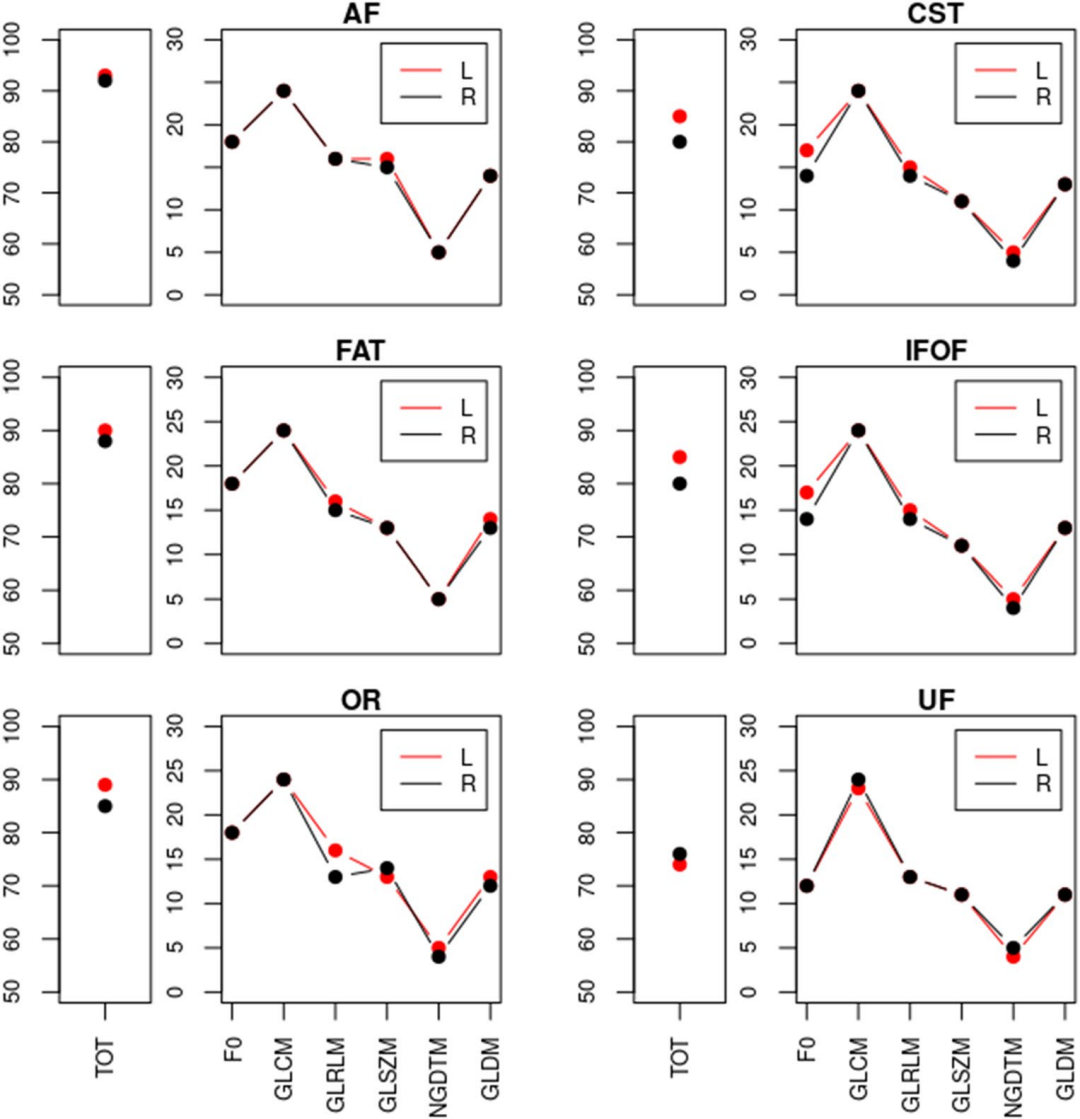
Robustness sub-analysis by side. Line plot with the number of robust features in NAWM tracts, divided into the different categories, for the left (L, red) and the right (R, black) side. Features were extracted setting 64 as binning and ICC was evaluated between the 4 (using different echo times) QSM reconstructions, setting 0.85 as threshold level (NAWM = normal appearing white matter, ICC = intraclass correlation coefficient, QSM = quantitative susceptibility mapping, AF = arcuate fasciculus, CST = cortico-spinal tract, FAT = frontal aslant tract, IFOF = inferior fronto-occipital fasciculus, OR = optic radiation, UF = uncinate fasciculus, FO = first-order, GL = grey level, GLCM = GL co-occurrence matrix, GLRLM = GL run length matrix, GLZM = GL zone matrix, NGTDM = neighbouring gray tone difference matrix, GLDM = GL dependence matrix).

To discover possible differences between the left and right hemispheres, the two were compared for each VOI. Results for NAWM tracts are reported in Fig. 6: the left hemisphere showed slightly higher robustness; however, differences between the two sides were negligible (*p* > 0.05). The same results were obtained for the overall NAWM (*p* > 0.05). The ICC matrix for this sub-analysis is shown in the Supplementary Materials (Supplementary Figure S9).

### Robust features across all VOIs

Several NAWM VOIs were analysed in this study, considering left and right hemispheres independently. Below, a summary of features that were found to be reliable in all the analysed ROIs:

- FO (50%, 9/18): 10th and 90th percentile, energy and total energy, mean, median and RMS, skewness and kurtosis (Supplementary Table S2)
- GLCM (~ 80%, 19/24): cluster shade, contrast, correlation, difference average, difference entropy, difference variance, inverse difference, inverse difference moment, inverse different moment normalised, inverse difference normalised, information measure of correlation 1 and 2, inverse variance, joint average, joint energy, joint entropy, maximal correlation coefficient, maximum probability and sum average (Supplementary Table S4)
- GLRLM (~ 65%, 10/16): GL non uniformity, long run emphasis, long run high GL emphasis, run entropy, run length non uniformity, run length non uniformity normalised, run percentage, run variance, short run emphasis, short run high GL emphasis (Supplementary Table S5)
- GLSZM (25%, 4/16): GL non uniformity, size zone non uniformity, zone entropy and zone percentage (Supplementary Table S6)
- NGDTM (80%, 4/5): busyness, coarseness, contrast, strength (Supplementary Table S7)
- GLDM (~ 65%, 9/14): dependence entropy, dependence non-uniformity, dependence non uniformity normalised, dependence variance, GL non uniformity, large dependence emphasis, large dependence high GL emphasis, small dependence emphasis, small dependence high GL emphasis (Supplementary Table S8)

Features were evaluated first by considering together left and right hemispheres and then by splitting the two sides. In the Supplementary Tables S16 to S18 ICC values of robust features for left and right hemispheres together and individually are reported. 65% of radiomic features were robust within the entire NAWM with no fewer than 50% in each class. Texture classes showed higher robustness (NGTDM 80%, GLCM 79.2%, GLDM 71.4%, GLRLM 62.5%, GLSZM 56.3%), compared to first-order statistics (50%). Analysis of WM tracts showed that more than 75% of features were robust within all the analysed NAWM tracts (Fig. 3). Texture classes GLCM (99.3%) and GLRLM (96.9%) showed a higher percentage of robust features compared to GLDM (91.7%), NGTM (90.0%), FO (83.3%) and GLSZM (81.3%).

## Discussion

This study demonstrated that QSM-derived radiomics features assessed within the NAWM of patients with MS and healthy controls, both overall and within selected tracts, are robust, albeit with some degree of variability among different structures. We explored potential factors that impacted robustness, to help in future selection of anatomical VOIs/ROIs, and identified the subset of robust radiomic features that warrants future investigation as potential aid in clinical decision making. For the analysis, we considered a mixed sample of patients and healthy controls focusing on the non-lesioned white matter tissue to assess features’ reliability regardless of clinical condition; future work will help find differences in radiomic measurements between controls and patients and different clinical phenotypes.

Conventional MR images have arbitrary (non-calibrated) grey intensity values, thus, they require image normalisation prior to undergo feature extraction. Different methods have been proposed for image normalisation, and the chosen method may have a direct effect on the predictivity of the features obtained from the normalised images^56^. In our study, we used an intrinsically quantitative sequence, and all the exams were acquired using the same scanner and processed with a locked-down pipeline, generating a uniquely homogenous dataset. Hence, we avoided normalisation of intensity histograms that is often necessary when analysing heterogeneous medical images^25^.

Intensity values on QSM images quantitatively express the underlying magnetic susceptibility within a given voxel; changing visualisation parameters affects how the images are perceived due to the limited perception of GLs by the human eye (higher number of GLs corresponding to higher contrast resolution), but does not influence the quantitative value expressing the magnetic susceptibility of the examined structure. Hence, we firstly explored how different numbers of GLs (bin number) influence robustness of radiomic features keeping the bin size fixed. This approach is recommended by the Image Biomarker Standardisation Initiative^28^ when dealing with quantitative data as it maintains a direct relationship with the original intensity value.

In our study, the number of robust features was hovering above 525 for GLs values higher than 16, peaking at 64 GLs (555 features), hence, this was chosen as the optimal level for quantisation of QSM and was used for the ensuing robustness analysis. This result held when each size was analysed independently. Interestingly, a recent study exploring robustness of radiomic features on computed tomography images of liver and muscles, also a quantitative imaging modality, achieved similar results, with the optimal range of GLs determined at 32–64^30^, confirming that GLs need to be ample enough to cater for differences in neighbouring pixels and produce meaningful results.

Previous studies demonstrated that the size of the VOI can significantly influence radiomic features^57, 58^. First-order features are confounded by large volumes due to the higher number of voxels within the VOI^59^; thus, as NAWM was the largest VOI in our analysis, we expected a higher degree of variability and this was experimentally confirmed. A recent study assessing the reproducibility of QSM-derived radiomic features in dissecting intramural hematomas and atherosclerotic calcifications of intracranial vertebral arteries also observed volume-dependence of some features with the small volume of the VOIs jeopardising robustness^54^.

This information may have particular relevance when comparing NAWM of patients with MS and healthy controls as accelerated brain volume loss is a known phenomenon in patients with MS potentially resulting in a significant confounding factor due to the resulting different size of the NAWM among the two groups. Ideally, VOIs of dissimilar size should not be compared if the chosen metrics are sensitive to volume-effect. If excluding metrics sensitive to volume-effect cannot be avoided, such as in this specific context, a normalisation for volume dependency must be implemented in the pipeline, when VOIs have different sizes^30, 57, 60^.

Despite their sensitivity to volume effects, tract-based analysis in our cohort showed that most of the FO statistic descriptors (10th and 90th percentile, mean, median, skewness, and kurtosis) were robust in all the examined tracts, even on side-based sub-analysis, and performed better than texture descriptors. FO statistic descriptors are related to tissue bulk susceptibility and are directly proportional to biomarker concentration: susceptibility values correlate, positively and negatively respectively, with iron and myelin concentration^8^, both altered in several neurological disorders, including MS. The robustness of FO statistic descriptors is consistent with recent studies demonstrating high reproducibility of susceptibility values across different sites^61, 62^ suggesting the suitability of this technique for multicentre, cross-vendor, quantitative analyses of brain iron and myelin.

Examining the individual tracts statistics, our analysis undeniably split the NAWM tracts into two clusters: AF, FAT, and OR were more robust (with 100%, 96.8%, and 95.7% robust features respectively), and CST, IFOF, and UF were less robust (with 84.9%, 91.4%, and 77.4% of robust features respectively). As previously discussed, the volume of the VOI influences radiomics features, indeed, reviewing the volume distribution among those tracts (Fig. 4) confirmed that CST and IFOF had the largest volume among the examined white matter tracts. Nevertheless, the volume-dependence does not explain the performance of the UF, whose volume was not larger than that of AF or FAT.

Compelling evidence from the variance map built using QSM images of the healthy volunteers showed that along the CST, IFOF and UF voxels were more variable, hence, radiomic features would be less reliable. Moreover, the variance map clearly showed that regions with more variability were adjacent to areas prone to streak artifacts, for instance, closer to the air-tissue boundaries or cortical bone and/or close to high susceptibility sources^63, 64^, such as the basal ganglia, known to have age-related deposition of susceptibility-inducing compounds^65, 66^. Although reconstructed tracts should not overlap those areas, stability of features based on GL intensity may be influenced by their proximity. Indeed, susceptibility variance of both IFOF and UF peaked in the anterior cranial fossa, particularly prone to streaking artifacts. The UF is possibly more negatively influenced due to its smaller volume resulting in the number of unreliable pixels being a higher overall proportion.

Of distinct interest in the clinical context of MS, texture features in the CST revealed the underlying microstructural properties of the tract itself (Table 2). Entropy was lower in the CST compared to other tracts (Fig. 4), implying that the randomness in susceptibility values was lower. The uniformity distribution within the CST was higher compared to other tracts (Fig. 4), implying greater homogeneity. A plausible explanation for those findings would be that WM fibres in the CST are directed cranio-caudally, while the other tracts have a combination of fibres with heterogeneous directions: hence, we can infer that the uniform orientation results in uniformity of texture features. Motor symptoms have a high prevalence in patients with MS and cause significant deterioration in patients' quality of life and self-reliance. The robustness of radiomics features in the CST may lead to the development of a radiomic signature of the CST that could be prospectively monitored to detect early changes in the pyramidal tract before symptoms ensue and lesions become apparent on morphological imaging.

QSM is normally acquired using a variable number of echo times, although there is currently no consensus on number and value of TEs. Our study demonstrated an appreciable agreement between results extracted from susceptibility maps obtained with different TE, including maps generated with a single TE (47.1 ms). However, although this may suggest that performing radiomics on a limited number of TE would be sufficient, such an approach would result in loss of QSM-derived information that may provide different input. For instance, short echo times improve the visualisation of fine details aiding in the visualisation of small demyelinating lesions in patients with MS. Moreover, short and long echo times help to balance image contrast and image degradation by streak artifacts. Lastly, the use of multiple number of echo times leads to high SNR for multiple tissues and structures in the brain^52^. Hence, using multiple TEs benefits both the stability of the radiomics analysis and the conventional assessment of the derived radiological images.

Few recent studies have explored the application of radiomics to QSM images. Xiao et al*.*^32^ built a machine learning algorithm able to distinguish patients with Parkinson’s disease (PD) and HCs using radiomics features extracted from the substantia nigra (SN). More recently, Kang et al*.*^34^ compared radiomics features extracted from SN, head of caudate nucleus, and putamen among patients with PD and HCs confirming that features extracted from the SN performed better in diagnosing PD. Both studies focused on small, albeit clinically relevant, ROIs and did not provide any formal assessment of reproducibility or repeatability of their results.

Zhang et al*.*^33^ implemented a deep convolutional neural network fusing lesion-level radiomic and convolutional image features for automated identification of chronic active MS lesions (QSMRim-Net) as defined by the presence of a peripheral ‘rim’ of iron-laden activated microglia/macrophages. QSMRim-Net showed promising results in detecting chronic active lesions, however, the training and validation dataset was relatively small, while rim positive lesions were relatively scarce. Similarly, Yan et al*.*^31^ built a machine learning model combining radiomic features extracted from deep grey matter regions and demographic characteristics able to differentiate patients with MS and neuromyelitis optica spectrum disorder (NMOSD) with high accuracy. The model used only image information from deep grey matter structures that are not characteristically involved in NMOSD, but rather associated with antibodies against myelin oligodendrocyte glycoprotein (MOG-IgG)–positive status; this status was not disclosed by the authors, hence, raising the possibility of overestimating the capability of distinguishing between the two disorders^67^. The choice of using deep grey matter regions may have been dictated by the paucity of lesions with rim-like paramagnetic phase changes detectable in patients with NMOSD^68^. However, the limitations of the deployed strategy remain valid and question the capacity of this model to distinguish between NMOSD and MS.

Despite the limitations of those studies, there is an emerging potential application for sophisticated analytical approaches of QSM data in neurodegenerative and neuroinflammatory disorders. The robustness assessment described in this work could significantly contribute to future studies. For instance, both NMOSD and MS are known to affect the CST; our study highlighted the set of robust features that could be reliably used to assess changes in the CST, paving the way for a potential computer-assisted distinction between those two disorders. Moreover, although we did not assess microstructural information in this study, the ability to assess DWI is embedded in the pipeline, permitting the integration of susceptibility and diffusion measurements.

### Limitations

This study has a few limitations. First, although the size of the sample recruited was large, ultimately the patient subgroup was noticeably larger than the control group, due to the hybrid clinical/research scope of our institution. To control for this limitation, we performed a sub-group analysis in patient and control groups, confirming that independent results were consistent with the pooled analysis. This information may be helpful in guiding sample size selection for future studies (e.g., disease characterization), as obtaining a similar number of patients and controls may be challenging.

Second, our sample is drawn from a single site, and scans were all performed using the same clinical scanner (single-site, single-vendor setting). Although this improved the homogeneity of the data, there is a risk that radiomic features identified as robust in this study may be specific to datasets derived with similar scanners and in similar settings. Testing robustness on a more heterogeneous dataset would allow for a more thorough assessment of robustness decoupling the results from the acquisition and post-processing techniques. However, now that we have identified robust radiomic features in this specific setting, the proposed analytical pipeline can be used for replication studies to confirm reproducibility of our results using different hardware manufacturers and in different settings.

Last, we chose to use an automatic tool for lesion segmentation to ensure the same level of accuracy and potential bias throughout the analysis, avoiding intra- and inter- subject variability that necessarily occurs using manual segmentation^69^. This approach ensured that the segmentation would not influence our results, however, automatic lesion segmentation tools have limited accuracy for small lesions (< 5 mm) potentially retaining those within what we labelled as ‘normal appearing’ tissue.

## Conclusion

In conclusion, to the best of our knowledge, this is the first study to assess robustness of QSM-derived radiomic features in NAWM. Image perturbation was performed changing the number of grey levels in the image and the number of echo times used to reconstruct quantitative maps, variables of interest for feature reliability evaluation. We demonstrated that more than 65% of features were robust in the entire NAWM. The white matter tracts showed higher robustness, with more than 75% of features robust in all the white matter tracts. Differences among tracts are deemed to be secondary to the volume of the structure examined and the susceptibility variability distribution that may be influenced by nearby structures.

This work paves the way for future studies using the set of robust features we identified to non-invasively phenotype patients with MS, rapidly detect therapy response, and monitor the course of the disease.

### Supplementary Information

Supplementary Information.

## Data Availability

The data set generated and analysed during the current study are available in the Zenodo repository (https://zenodo.org/record/8271881).

## Abbreviations

AF: Arcuate fasciculus
BET: Brain extraction tool
CE-AVEC: “Area Vasta Emilia Centro” Ethics Committee
CNS: Central nervous system
CST: Cortico-spinal tract
DWI: Diffusion weighted imaging
FA: Flip angle
FAT: Fronto aslant tract
FLAIR: FLuid attenuate inversion recovery
FLIRT: FSL’s linear image registration tool
FMRIB: Functional magnetic resonance imaging of the brain
FO: First order
FSL: FMRIB software library
GL: Grey level
GLCM: GL co-occurrence matrix
GLDM: GL dependence matrix
GLRLM: GL run length matrix
GLZM: GL zone matrix
GRE: GRadient echo
HC: Healthy controls
ICC: Intra-class correlation coefficient
IFOF: Inferior fronto-occipital fasciculus
iLSQR: Iterative least square
LST: Lesion segmentation tool
MNI: Montreal Neurological Institute (MNI)
MOG-IgG: Antibodies against myelin oligodendrocyte glycoprotein
MPRAGE: Magnetization prepared RApid gradient echo
MRI: Magnetic resonance imaging
MS: Multiple sclerosis
NAWM: Normal appearing WM
NGTDM: Neighbouring grey tone difference matrix
NMOSD: Neuromyelitis optica spectrum disorder
OR: Optic radiation
PD: Parkinson’s disease
qMRI: Quantitative MRI
QSM: Quantitative susceptibility mapping
ROI: Region of interest
SN: Substantia nigra
SNR: Signal-to-noise ratio
SPM: Statistical parametric mapping
T_1_w: T_1_-weighted
T_2_w: T_2_-weighted
TE: Echo time
TR: Repetition time
UF: Uncinate fasciculus
V-SHARP: Variable kernel sophisticated harmonic artifact reduction for phase data
VOI: Volume of interest
WM: White matter
