# Supplementary material for: Assessing robustness of quantitative susceptibility-based MRI radiomic features in patients with multiple sclerosis
Source: Sci Rep. 2023 Sep 27;13:16239. doi: 10.1038/s41598-023-42914-4 (PMC10533494; doi:10.1038/s41598-023-42914-4)
Supplement: Supplementary file 1 — Supplementary Information. [file 41598_2023_42914_MOESM1_ESM.pdf]

Supplementary Materials

Abbreviations

AF = Arcuate Fasciculus
BET = Brain Extraction Tool
CE-AVEC = "Area Vasta Emilia Centro" Ethics Committee
CNS = Central Nervous System
CST = Cortico-Spinal Tract
DWI = Diffusion Weighted Imaging
FA = Flip Angle
FAT = Fronto Aslant Tract
FLAIR = FLuid Attenuate Inversion Recovery
FLIRT = FSL's Linear Image Registration Tool
FMRIB = Functional Magnetic Resonance Imaging of the Brain
FO = First Order
FSL = FMRIB Software Library
GL = Grey Level
GLCM = GL Co-occurrence Matrix
GLDM = GL Dependence Matrix
GLRLM = GL Run Length Matrix
GLZM = GL Zone Matrix
GRE = GRadient Echo
HC = Healthy Controls
ICC = Intra-class correlation coefficient
IFOF = Inferior Fronto-Occipital Fasciculus
iLSQR = iterative least square
LST = Lesion Segmentation Tool
MNI = Montreal Neurological Institute (MNI)
MOG-IgG = antibodies against myelin oligodendrocyte glycoprotein
MPRAGE = Magnetization Prepared RApid Gradient Echo
MRI = Magnetic Resonance Imaging
MS = Multiple Sclerosis
NAWM = Normal Appearing WM
NGTDM = Neighbouring Grey Tone Difference Matrix
NMOSD = Neuromyelitis Optica Spectrum Disorder
OR = Optic Radiation
PD = Parkinson's Disease
qMRI = quantitative MRI
QSM = Quantitative Susceptibility Mapping
ROI = Region of Interest
SN = Substantia Nigra
SNR = Signal-to-Noise Ratio
SPM = Statistical Parametric Mapping
T_1w = T_1-weighted
T_2w = T_2-weighted
TE = Echo Time
TR = Repetition Time
UF = Uncinate Fasciculus
V-SHARP = Variable kernel Sophisticated Harmonic Artifact Reduction for Phase data
VOI = Volume of Interest
WM = White Matter

Materials and Methods

MR Protocol

	MPRAGE 3D T_1w	FLAIR 3D SPACE T_2w	HARDI DWI 2D EPI single-shot	QSM 3D GRE T_2*w
**Plane**	sagittal	sagittal	axial	axial
**TR (ms)**	2300	5000	4300	53
**TE (ms)**	2.98	428	98	9.42
**ΔTE (ms)**	NA	NA	NA	9.42
**N° TEs**	1	1	1	5
**SR (mm^3)**	1x1x1	1x1x1	2x2x2	0.5x0.5x1.5
**FA (°)**	9	120	90	15
**Scan time**	~ 5’	~ 6’	~ 9’	~ 9’

Table S1: MR protocol (MPRAGE = Magnetization Prepared RApid Gradient Echo; FLAIR = FLuid Attenuated Inversion Recovery; SPACE = Sampling Perfection with Application optimised Contrast using different flip angle Evolution; HARDI = High Angular Resolution Diffusion Imaging; DWI = Diffusion Weighted Imaging; EPI = Echo Planar Imaging; QSM = Quantitative Susceptibility Mapping; GRE = Gradient-echo; TR = Time of Repetition; TE = Time of Echo; SR = Spatial Resolution; FA = Flip Angle; NA = Not Available)

For DWI measurements, single-shell (5 unweighted vols and 60 vols b = 2000 s/mm^2) and multi-shell (8 unweighted vols, 8 vols b = 300 s/mm^2, 30 vols b = 1000 s/mm^2 and 64 vols b = 2000 s/mm^2) sequences were used, with Anterior-Posterior (AP) phase encoding, and an additional sequence with inverted phase encoding (PA) (~ 4’ scan time) to correct Echo-Planar Imaging (EPI) distortion artifacts, as in [43] and [50].

WM Tracts

The analysed WM tracts were: **Arcuate Fasciculus (AF)**, connecting the temporal and the inferior parietal cortex to locations in the frontal lobe and linking Broca’s and Wernicke’s areas (language prediction and understanding); **Cortico-Spinal Tract (CST)**, also known as pyramidal tract, is the major neuronal pathway providing voluntary motor function, connecting the cortex to the spinal cord to enable movement of the distal extremities; **Frontal Aslant Tract (FAT)** connects the superior frontal gyrus (pre-SMA Supplementary Motor Area) and SMA to the pars opercularis and pars triangularis of the inferior frontal gyrus and the anterior insula, it is strongly associated with speech initiation, verbal fluency and stuttering; **Inferior Fronto-Occipital Fasciculus (IFOF)**, large white matter tract associated with semantic language processing and goal-oriented behaviour; **Optic Radiation (OR)**, known as geniculocalcarine tract, connects the lateral geniculate nucleus to the primary visual cortex in the occipital lobe (transmission of the visual input passing through the retina, the optic nerve and the optic tract); **Uncinate Fasciculus (UF)** links the orbitofrontal cortex, involved in face encoding and in processing famous names, to the temporal pole, which is crucial in naming people.

Radiomic Features

Below, is a comprehensive list of the features extracted with Pyradiomics [51] (https://pyradiomics.readthedocs.io/en/latest/features.html), including their definition and explanation. They were divided into classes: First Order (FO), Shape 3D (S3D), Grey Level Co-

occurrence Matrix (GLCM), Grey Level Run Length Matrix (GLRLM), Grey Level Size Zone Matrix (GLSZM), Neighbouring Grey Tone Difference Matrix (NGTDM) and Grey Level Dependence Matrix (GLDM).

First order features

X : set of N_p voxels included in the ROI
N_p: number of non-zero bins
$P(i)$: first order histogram with N_g discrete intensity level
$p(i)$: normalised first order histogram $\frac{P(i)}{N_p}$
c: optimal value which shifts the intensities to prevent negative values in X
ϵ : arbitrary small positive number (~ 2.2 x 10^{-16})

1	10th P	10th percentile of X	---
2	90th P	90th percentile of X	---
3	Energy	$\sum_{i=1}^{N_p}(X(i)+c)^2$	It measures the magnitude of voxel values in an image, larger values mean a greater sum of the square of these values
4	Entropy	$-\sum_{i=1}^{N_p}p(i)\,(p(i)+\epsilon)$	It measures the uncertainty/randomness in the image values
5	Interquartile Range	75th P – 25th P	25th and 75th percentile of X
6	Kurtosis	$\frac{\mu_4}{\sigma_4}=\frac{\frac{1}{N_p}\sum_{i=1}^{N_p}(X(i)-\underline{X})^4}{(\sqrt{\frac{1}{N_p}\sum_{i=1}^{N_p}(X(i)-\underline{X})^2}^{\,2}}$	It measures the ‘peakedness’ of the distribution of values
7	Maximum	$max\,(X)$	Maximum value of the histogram of intensities
8	Mean Absolute Deviation (MAD)	$\frac{1}{N_p}\sum_{i=1}^{N_p}\lvert X(i)-\underline{X}\rvert$	Mean distance of all intensity values from the mean value of the image array
9	Mean	$\frac{1}{N_p}\sum_{i=1}^{N_p}X(i)$	---
10	Median	50th percentile of X	---
11	Minimum	$min\,(X)$	Minimum value of the histogram of intensities
12	Range	$max\,(X)-min\,(X)$	---
13	Robust MAD	$\frac{1}{N_{10-90}}\sum_{i=1}^{N_{10-90}}\lvert X_{10-90(i)}-\underline{X_{10-90}}\rvert$	MAD calculated on the subset of image array with GLs between the 10th and the 90th percentile
14	Root Mean Squared	$\sqrt{\frac{1}{N_p}\sum_{i=1}^{N_p}(X(i)+c)^2}$	It measures the magnitude of the image values; it is volume-confounded
15	Skewness	$\frac{\mu_3}{\sigma_3}=\frac{\frac{1}{N_p}\sum_{i=1}^{N_p}(X(i)-\underline{X})^3}{(\sqrt{\frac{1}{N_p}\sum_{i=1}^{N_p}(X(i)-\underline{X})^2}^{\,3}}$	It measures the asymmetry of the distribution of values with respect to the mean value

16	Total Energy	$V_{voxel}\sum_{i=1}^{N_p}(X(i)+c)^2$	Total Energy is the Energy scaled by the volume (mm^3)
17	Uniformity	$\sum_{i=1}^{N_g}p(i)^2$	It measures the homogeneity of the image array, where a greater uniformity implies a greater homogeneity or a smaller range of discrete intensity values
18	Variance	$\frac{1}{N_p}\sum_{i=1}^{N_p}(X(i)-\underline{X})^2$	It measures the spread of the distribution around the mean

Table S2: List of First Order (FO) features

Shape 3D features

N_v: number of voxels included in the ROI
N_f: number of faces (triangles) defining the Mesh
V : volume of the mesh in mm^3
A : surface area of the mesh in mm^2

1	Elongation	$\sqrt{\frac{\lambda_{minor}}{\lambda_{major}}}$	$[0,1]$, where 0 = the object is maximally elongated and 1 = the cross-section through the first and the second largest principal moment is circle-like
2	Flatness	$\sqrt{\frac{\lambda_{least}}{\lambda_{major}}}$	$[0,1]$, where 0 = flat object and 1 = non-flat object, sphere like
3	Least Axis Length	$4\sqrt{\lambda_{least}}$	It yields the smallest axis length of the ROI-enclosing ellipsoid and is calculated using the largest principal component
4	Major Axis Length	$4\sqrt{\lambda_{major}}$	It yields the largest axis length of the ROI-enclosing ellipsoid and is calculated using the largest principal component
5	Maximum 2D Diameter Column	Largest pairwise Euclidean distance between surface mesh vertices in the row-slice plane	Usually, coronal plane
6	Maximum 2D Diameter Row	Largest pairwise Euclidean distance between surface mesh vertices in the column-slice plane	Usually, sagittal plane
7	Maximum 2D Diameter Slice	Largest pairwise Euclidean distance between surface mesh vertices in the row-column plane	Usually, axial plane
8	Maximum 3D Diameter	Largest pairwise Euclidean distance between surface mesh vertices	Feret Diameter

9	Mesh Volume	$\sum_{i=1}^{N_f} V_i = \sum_{i=1}^{N_f} \frac{Oa_i \cdot (Ob_i \times Oc_i)}{6}$	Evaluated from the triangle mesh of the ROI
10	Minor Axis Length	$4\sqrt{\lambda_{minor}}$	It yields the second-largest axis length of the ROI-enclosing ellipsoid and is calculated using the largest principal component
11	Sphericity	$\frac{\sqrt[3]{36\pi V^2}}{A}$	$(0,1]$ with 1 = perfect sphere
12	Surface Area	$\sum_{i=1}^{N_f} A_i = \sum_{i=1}^{N_f} \frac{1}{2}\lvert a_i b_i \times a_i c_i \rvert$	$a_i b_i$ and $a_i c_i$ are the edges of the i^{th}triangle in the mesh, with vertices a_i, b_i and c_i. Area of each triangle in the mesh is calculated and then the total surface area is obtained.
13	Surface Volume Ratio	$\frac{A}{V}$	A lower value indicates a more compact (sphere-like) shape
14	Voxel Volume	$\sum_{k=1}^{N_v} V_k$	Approximating by multiplying the number of voxels in the ROI by the volume of a single voxel

Table S3: List of Shape 3D (S3D) features

Grey Level Co-occurrence Matrix features

A Grey Level Co-occurrence Matrix (GLCM) ($N_g \times N_g$, whereN_g is the number of discrete intensity levels in the image) is a second-order joint probability function defined as $P(i,j|\delta,\theta)$, counting the number of times the combination of the ith and the jth level, separated by a distance δ along an angle θ occurs. Pyradiomics computes symmetrical GLCM by default.

ϵ : arbitrary small positive number (~ 2.2 x 10^{-16})
$P(i,j)$: co-occurrence matrix for an arbitrary δ and θ
$p(i,j)$: normalised co-occurrence matrix equal to $\frac{P(i,j)}{\sum P(i,j)}$
N_g : number of discrete intensity levels in the image
$p_x(i) = \sum_{j=1}^{N_g} p(i,j)$: marginal row probabilities
$p_y(j) = \sum_{i=1}^{N_g} p(i,j)$: marginal column probabilities
μ_x: mean grey level intensity of p_x defined as $\sum_{i=1}^{N_g} p_x(i)i$
μ_y: mean grey level intensity of p_y defined as $\sum_{j=1}^{N_g} p_y(j)j$
σ_x: standard deviation of p_x
σ_y: standard deviation of p_y
$p_{x+y}(k) = \sum_{i=1}^{N_g}\sum_{j=1}^{N_g} p(i,j)$ where $i + j = k$ and $k = 2,3,\dots,2N_g$
$p_{x-y}(k) = \sum_{i=1}^{N_g}\sum_{j=1}^{N_g} p(i,j)$where $|i-j| = k$ and $k = 0,1,\dots,N_g$
$HX = -\sum_{i=1}^{N_g} p_x(i)(p_x(i) + \epsilon)$: entropy of p_x
$HY = -\sum_{j=1}^{N_g} p_y(j)(p_y(j) + \epsilon)$: entropy of p_y

$HXY = -\sum_{i=1}^{N_g}\sum_{j=1}^{N_g} p(i,j)(p(i,j)+\epsilon)$: entropy of $p(i,j)$

$$HXY1 = -\sum_{i=1}^{N_g}\sum_{j=1}^{N_g} p(i,j)(p_x(i)p_y(j)+\epsilon)$$

$$HXY2 = -\sum_{i=1}^{N_g}\sum_{j=1}^{N_g} p_x(i)p_y(j)(p_x(i)p_y(j)+\epsilon)$$

1	Autocorrelation	$\sum_{i=1}^{N_g}\sum_{j=1}^{N_g} p(i,j)ij$	Measure of the fineness and the coarseness of texture
2	Cluster Prominence	$\sum_{i=1}^{N_g}\sum_{j=1}^{N_g} (i+j-\mu_x-\mu_y)^4 p(i,j)$	Measure of skewness and asymmetry of the GLCM (higher value: more asymmetry)
3	Cluster Shade	$\sum_{i=1}^{N_g}\sum_{j=1}^{N_g} (i+j-\mu_x-\mu_y)^3 p(i,j)$	—
4	Cluster Tendency	$\sum_{i=1}^{N_g}\sum_{j=1}^{N_g} (i+j-\mu_x-\mu_y)^2 p(i,j)$	Measure of groupings of voxels with similar GL values
5	Contrast	$\sum_{i=1}^{N_g}\sum_{j=1}^{N_g} (i-j)^2 p(i,j)$	Local intensity variation; the larger is, the grater is the disparity in intensity values among neighbouring voxels
6	Correlation	$\frac{\sum_{i=1}^{N_g}\sum_{j=1}^{N_g} p(i,j)ij - \mu_x\mu_y}{\sigma_x(i)\sigma_y(j)}$	From 0 (= uncorrelated) and 1 (perfectly correlated), it shows the linear dependency of grey level values to their respective voxels in the GLCM
7	Difference Average	$\sum_{k=0}^{N_g-1} kp_{x-y}(k)$	Relationship between occurrences of pairs with similar intensity values and occurrences of pairs with different intensity values
8	Difference Entropy	$\sum_{k=0}^{N_g-1} p_{x-y}(k)(p_{x-y}(k)+\epsilon)$	It measures the randomness/variability in the neighbourhood intensity value difference
9	Difference Variance	$\sum_{k=0}^{N_g-1} (k-DA)^2 p_{x-y}(k)$	It measures heterogeneity, setting higher weights on different intensity level pairs that deviate more from the mean
10	Id (Inverse difference)	$\sum_{k=0}^{N_g-1} \frac{p_{x-y}(k)}{1+k}$	(homogeneity 1)<br>Another measure of local homogeneity (higher with more uniform grey level)
11	Idm (Id moment)	$\sum_{k=0}^{N_g-1} \frac{p_{x-y}(k)}{1+k^2}$	Measure of the local homogeneity of an image (weights opposite to the contrast)
12	Idmn (Idm normalised)	$\sum_{k=0}^{N_g-1} \frac{p_{x-y}(k)}{1+\frac{k^2}{N_g^2}}$	Measure of the local homogeneity of an image (weights opposite to the contrast)
13	Idn (Id normalised)	$\sum_{k=0}^{N_g-1} \frac{p_{x-y}(k)}{1+\left(\frac{k}{N_g}\right)}$	Another measure of homogeneity in an image

14	Imc1 (Information measure of correlation 1)	$\frac{HXY - HXY1}{max\{HX, HY\}}$	—
15	Imc2 (Information measure of correlation 2)	$\sqrt{1 - e^{-2(HXY2 - HXY)}}$	—
16	Inverse Variance	$\sum_{k=0}^{N_g-1} \frac{p_{x-y}(k)}{k^2}$	—
17	Joint Average	$\mu_x = \sum_{i=1}^{N_g} \sum_{j=1}^{N_g} p(i,j)i$	Returns the mean GL intensity of the ith distribution
18	Joint Energy	$\sum_{i=1}^{N_g} \sum_{j=1}^{N_g} (p(i,j))^2$	Measure of homogeneous patterns in the image
19	Joint Entropy	$-\sum_{i=1}^{N_g} \sum_{j=1}^{N_g} p(i,j)(p(i,j) + \epsilon)$	Measure of variability in the neighbourhood intensity values
20	MCC	$\sqrt{second\ largest\ eigenvalue\ of\ Q}$ $Q(i,j) = \sum_{k=0}^{N_g} \frac{p(i,k), p(j,k)}{p_x(i) p_y(k)}$	Maximal Correlation Coefficient, measure of complexity of the texture [0,1] (1 = flat region)
21	Maximum Probability	$max\ (p(i,j))$	Occurrences of the most predominant pair of neighbouring values
22	Sum Average	$\sum_{k=2}^{2N_g} p_{x+y}(k)k$	Occurrences of pairs with lower intensities with pairs with higher intensities
23	Sum Entropy	$\sum_{k=2}^{2N_g} p_{x+y}(k)(p_{x+y}(k) + \epsilon)$	Sum of neighbourhood intensity value differences
24	Sum Squares	$\sum_{i=1}^{N_g} \sum_{j=1}^{N_g} (i - \mu_x)^2 p(i,j)$	Or Variance, in the distribution neighbouring intensity level pairs

Table S4: List of Grey Level Co-occurrence Matrix (GLCM) features

Grey Level Run Length Matrix

A Grey Level Run Length Matrix (GLRLM) quantifies grey level runs, which are the length in numbers of consecutive pixels with the same grey value; the element $P(i,j|\theta)$ describes the number of runs with a grey level i and length j in the image along an angle θ.
By default, the value of each feature is evaluated for each θ individually and then different values are averaged.

ϵ : arbitrary small positive number (~ 2.2 x 10^{-16})
N_g : number of discrete intensity levels in the image
N_r : number of discrete run lengths in the image
N_p : number of voxels in the image
$N_r(\theta)$: number of runs in the image along angle θ equal to $\sum_{i=1}^{N_g} \sum_{j=1}^{N_r} P(i,j|\theta)$ and $1 \leq N_r(\theta) \leq N_p$
$P(\theta)$: run length matrix for an arbitrary direction θ
$p(\theta)$: normalised run length matrix, defined as $p(\theta) = \frac{P(\theta)}{N_r(\theta)}$

1	GL Non-Uniformity (GLN)	$\frac{\sum_{i=1}^{N_g} (\sum_{j=1}^{N_r} P(\theta))^2}{N_r(\theta)}$	It measures the similarity of GL intensity value in the image

			(lower = greater similarity in intensity values)
2	GLN Normalised (GLNN)	$\frac{\sum_{i=1}^{N_g}(\sum_{j=1}^{N_r}P(\theta))^2}{N_r(\theta)^2}$	Normalised version of GLN
3	GL Variance (GLV)	$\sum_{i=1}^{N_g}\sum_{j=1}^{N_r}p(\theta)(i-\mu)^2,$ $\mu = \sum_{i=1}^{N_g}\sum_{j=1}^{N_r}p(\theta)i$	Variance in grey level intensity for the runs
4	High GL Run Emphasis (HGLRE)	$\frac{\sum_{i=1}^{N_g}\sum_{j=1}^{N_r}P(\theta)i^2}{N_r(\theta)}$	Measure of the distribution of the higher grey-level runs, higher values indicate a greater concentration of high grey level values in the image
5	Long Run Emphasis (LRE)	$\frac{\sum_{i=1}^{N_g}\sum_{j=1}^{N_r}P(\theta)j^2}{N_r(\theta)}$	Measure of the distribution of long run lengths, greater when there are longer lengths and more coarse structural textures
6	Long Run High GL Emphasis (LRHGLRE)	$\frac{\sum_{i=1}^{N_g}\sum_{j=1}^{N_r}P(\theta)i^2j^2}{N_r(\theta)}$	It measures the joint distribution of long run lengths with higher grey-level values
7	Long Run Low GL Emphasis (LRLGLE)	$\frac{\sum_{i=1}^{N_g}\sum_{j=1}^{N_r}\frac{P(\theta)j^2}{i^2}}{N_r(\theta)}$	It measures the joint distribution of long run lengths with lower grey-level values
8	Low GL Run Emphasis (LGLRE)	$\frac{\sum_{i=1}^{N_g}\sum_{j=1}^{N_r}\frac{P(\theta)}{i^2}}{N_r(\theta)}$	Measure of the distribution of the lower grey-level runs, higher values indicate a greater concentration of low grey level values in the image
9	Run Entropy (RE)	$-\sum_{i=1}^{N_g}\sum_{j=1}^{N_r}p(\theta)(p(\theta)+\epsilon)$	Measure of the uncertainty/randomness in the run lengths and grey level distribution: higher values indicate more heterogeneity in the texture patterns
10	Run Length Non-Uniformity (RLN)	$\frac{\sum_{j=1}^{N_r}(\sum_{i=1}^{N_g}P(\theta))^2}{N_r(\theta)}$	It measures the similarity of run lengths, with lower values indicating more homogeneity among run lengths
11	RLN Normalized (RLNN)	$\frac{\sum_{j=1}^{N_r}(\sum_{i=1}^{N_g}P(\theta))^2}{N_r(\theta)^2}$	Normalised version of RLN
12	Run Percentage (RP)	$\frac{N_r(\theta)}{N_p}$	$[\frac{1}{N_p},1]$ Measure of the coarseness of the texture (higher values indicate more fine texture)

13	Run Variance (RV)	$\sum_{i=1}^{N_g}\sum_{j=1}^{N_r} p(\theta)(j-\mu)^2$<br>$\mu = \sum_{i=1}^{N_g}\sum_{j=1}^{N_r} p(\theta)j$	Measure of the variance in runs for the run lengths
14	Short Run Emphasis (SRE)	$\sum_{i=1}^{N_g}\sum_{j=1}^{N_r} \frac{P(\theta)}{j^2}$	Measure of the distribution of short run lengths, greater when there are more short run lengths and more fine textural features
15	Short Run High GL Emphasis (SRHGLE)	$\frac{\sum_{i=1}^{N_g}\sum_{j=1}^{N_r}\frac{P(\theta)i^2}{j^2}}{N_r(\theta)}$	It measures the joint distribution of shorter run lengths with higher grey-level values
16	Short Run Low GL Emphasis (SRLGLE)	$\frac{\sum_{i=1}^{N_g}\sum_{j=1}^{N_r}\frac{P(\theta)}{i^2 j^2}}{N_r(\theta)}$	It measures the joint distribution of shorter run lengths with lower GL values

Table S5: List of Grey Level Run Length Matrix (GLRLM) features

Grey Level Size Zone Matrix

A Grey Level Size Zone Matrix (GLSZM) quantifies the grey level zones in an image, defined as the number of connected voxels that share the same intensity (distance = 1 according to the infinity norm – 36 connected regions in a 3D, 8-connected region in a 2D). In the matrix, the element $P(i,j)$ is the number of zones with grey level i and size j.

ϵ : arbitrary small positive number (~ 2.2 x 10^{-16})
N_g : number of discrete intensity levels in the image
N_s : number of discrete zone sizes in the image
N_p : number of voxels in the image
N_z : number of zones in the ROI, equal to $\sum_{i=1}^{N_g}\sum_{j=1}^{N_s} P(i,j)$and $1 \le N_z \le N_p$
$P(i,j)$: size zone matrix
$p(i,j)$: normalised size zone matrix, defined as $\frac{P(i,j)}{N_z}$

1	GL Non-Uniformity (GLN)	$\frac{\sum_{i=1}^{N_g}\left(\sum_{j=1}^{N_s} P(i,j)\right)^2}{N_z}$	Variability of GL intensity values in the image - lower value indicating more homogeneity in intensity values
2	GLN Normalised (GLNN)	$\frac{\sum_{i=1}^{N_g}\left(\sum_{j=1}^{N_s} P(i,j)\right)^2}{N_z{}^2}$	Normalised version of GLN
3	GL Variance (GLV)	$\sum_{i=1}^{N_g}\sum_{j=1}^{N_s} p(i,j)(i-\mu)^2$,<br>$\mu = \sum_{i=1}^{N_g}\sum_{j=1}^{N_s} p(i,j)i$	Variance in GL intensities for the zones
4	High GL Zone Emphasis (HGLZE)	$\frac{\sum_{i=1}^{N_g}\sum_{j=1}^{N_s} P(i,j)i^2}{N_z}$	It measures the distribution of higher grey-level size zones; high values

			indicate a greater proportion of higher grey-level values and size zones in the image
5	Large Area Emphasis (LAE)	$\frac{\sum_{i=1}^{N_g}\sum_{j=1}^{N_s}P(i,j)j^2}{N_z}$	Measure of the distribution of large area zones; greater values indicate bigger size zones and more coarse texture
6	Large Area High GL Emphasis (LAHGLE)	$\frac{\sum_{i=1}^{N_g}\sum_{j=1}^{N_s}P(i,j)i^2j^2}{N_z}$	It measures the proportion in the image of the joint distribution of larger size zones with higher GL values
7	Large Area Low GL Emphasis (LALGLE)	$\frac{\sum_{i=1}^{N_g}\sum_{j=1}^{N_s}\frac{P(i,j)j^2}{i^2}}{N_z}$	It measures the proportion in the image of the joint distribution of larger size zones with higher GL values
8	Low GL Zone Emphasis (LGLZE)	$\frac{\sum_{i=1}^{N_g}\sum_{j=1}^{N_s}\frac{P(i,j)}{i^2}}{N_z}$	It measures the distribution of lower grey-level size zones; high values indicate a greater proportion of lower grey-level values and size zones in the image
9	Size Zone Non-Uniformity (SZN)	$\frac{\sum_{j=1}^{N_s}(\sum_{i=1}^{N_g}P(i,j))^2}{N_z}$	It measures the variability of size zone volumes in the image, with a lower value indicating more heterogeneity in size zone volumes
10	SZN Normalized (SZNN)	$\frac{\sum_{j=1}^{N_s}(\sum_{i=1}^{N_g}P(i,j))^2}{N_z^2}$	Normalised version of SZN
11	Small Area Emphasis (SAE)	$\frac{\sum_{i=1}^{N_g}\sum_{j=1}^{N_s}\frac{P(i,j)}{j^2}}{N_z}$	Measure of the distribution of small size zones; greater values indicate smaller size zones and finer texture
12	Small Area High GL Emphasis (SAHGLE)	$\frac{\sum_{i=1}^{N_g}\sum_{j=1}^{N_s}\frac{P(i,j)i^2}{j^2}}{N_z}$	Measure of the proportion in the image of the joint distribution of smaller size zones with higher GL values
13	Small Area Low GL Emphasis (SALGLE)	$\frac{\sum_{i=1}^{N_g}\sum_{j=1}^{N_s}\frac{P(i,j)}{i^2j^2}}{N_z}$	Measure of the proportion in the image of the joint distribution of smaller size zones

			with lower GL values
14	Zone Entropy (ZE)	$-\sum_{i=1}^{N_g}\sum_{j=1}^{N_s} p(i,j)(p(i,j)+\epsilon)$	Randomness of the distribution of zone sizes and grey levels. Higher values indicate more heterogeneity in the texture patterns
15	Zone Percentage (ZP)	$\frac{N_z}{N_p}$	Measure of the coarseness of the texture - $[\frac{1}{N_p}, 1]$, with higher values indicating a larger portion of the ROI consists of small zones (finer texture)
16	Zone Variance (ZV)	$\sum_{i=1}^{N_g}\sum_{j=1}^{N_s} p(i,j)(j-\mu)^2$, $\mu = \sum_{i=1}^{N_g}\sum_{j=1}^{N_s} p(i,j)j$	Variance in zone size volumes for the zones

Table S6: List of Grey Level Size Zone Matrix (GLSZM) features

Neighbouring Grey Tone Difference Matrix

A Neighbouring Grey Tone Difference Matrix (NGTDM) is defined by the quantification of the difference between a grey value and the average grey values of its neighbours within distance δ. For each grey level i, the absolute difference in intensity is stored in the matrix.

X_{gl}: set of segmented voxels
$x_{gl}\,(j_x, j_y, j_z) \in X_{gl}$: grey level of a voxel at position $(j_x j_y j_z)$
The average grey level of the neighbourhood is:
$\bar{A}_i = \bar{A}(j_x, j_y, j_z) = \frac{1}{W}\sum_{k_z=-\delta}^{\delta}\sum_{k_y=-\delta}^{\delta}\sum_{k_z=-\delta}^{\delta} x_{gl}(j_x + k_x, j_y + k_y, j_z + k_z)$ where$(k_x, k_y, k_z) \neq (0,0,0)$ and $x_{gl}(j_x + k_x, j_y + k_y, j_z + k_z) \in X_{gl}$ and W is the number of voxels in the neighbourhood that are also in X_{gl}.
n_i: number of voxels in X_{gl} with grey level i
$N_{v,p}$: total number of voxels in $X_{gl}(\sum n_i)$
$N_{v,p} \leq N_p$, where N_p is the total number of voxels in the ROI
p_i: grey level probability equal to $\frac{n_i}{N_v}$
$s_i = \begin{cases} \sum_{n_i}|i - \underline{A}_i| \; for \; n_i \neq 0 \\ 0 \; for \; n_i = 0 \end{cases}$: sum of the absolute differences for grey level i

N_g: number of the discrete grey levels
$N_{g,p}$: number of the grey levels where $p_i \neq 0$

1	Busyness	$\frac{\sum_{i=1}^{N_g} p_i s_i}{\sum_{i=1}^{N_g}\sum_{j=1}^{N_g} \lvert ip_i - jp_j \rvert}$	A measure of change from a pixel to its neighbour. High value: 'busy' image, rapid changes in intensity

2	Coarseness	$\frac{1}{\sum_{i=1}^{N_g} p_i s_i}$	Measure of the average difference between the centre voxel and its neighbourhood; it indicates the spatial rate of change; higher values indicate a locally more uniform texture
3	Complexity	$\frac{1}{N_{v,p}}\sum_{i=1}^{N_g}\sum_{j=1}^{N_g}\lvert i-j\rvert\frac{p_i s_i + p_j s_j}{p_i + p_j}$	With many primitive components in the image: the image is non-uniform and there are many rapid changes in grey level intensity
4	Contrast	$\frac{1}{N_{g,p}(N_{g,p}-1)}\sum_{i=1}^{N_g}\sum_{j=1}^{N_g} p_i p_j (i-j)^2$	Measure of the spatial intensity change, depending also on the overall grey level dynamic range. Contrast is high: large range of grey levels, large changes between voxels and their neighbourhood
5	Strength	$\frac{\sum_{i=1}^{N_g}\sum_{j=1}^{N_g}(p_i + p_j)(i-j)^2}{\sum_{i=1}^{N_g} s_i}$	Measure of primitives in an image. Its value is high when the primitives are easily defined and visible

Table S7: List of Neighbouring Grey Tone Difference Matrix (NGTDM) features ($p_i \neq 0, p_j \neq 0$)

Grey Level Dependence Matrix

A Grey Level Dependence Matrix (GLDM) quantifies grey level dependencies in an image, defined as the number of connected voxels within a distance δ that are dependent on the centre voxel. A voxel with a grey level j is considered dependent on a centre voxel with a grey level i if $|i-j| \le \alpha$. $P(i,j)$ describes the number of times a voxel with GL i with j dependent voxels in its neighbourhood appear in image.

ϵ : arbitrary small positive number (~ 2.2 x 10^{-16})
N_g : number of discrete intensity levels in the image
N_d : number of discrete dependency sizes in the image
N_z : number of dependency zones in the image, equal to $\sum_{i=1}^{N_g}\sum_{j=1}^{N_d} P(i,j)$
$P(i,j)$: dependence matrix
$p(i,j)$: normalised dependence zone matrix, defined as $\frac{P(i,j)}{N_z}$

1	Dependence Entropy (DE)	$-\sum_{i=1}^{N_g}\sum_{j=1}^{N_d} p(i,j)(p(i,j)+\epsilon)$	---
2	Dependence Non-Uniformity (DN)	$\frac{\sum_{j=1}^{N_d}\left(\sum_{i=1}^{N_g} P(i,j)\right)^2}{N_z}$	Measure the similarity of dependence throughout the image; lower values indicate more homogeneity

			among dependencies
3	DN Normalised (DNN)	$\frac{\sum_{j=1}^{N_d}(\sum_{i=1}^{N_g}P(i,j))^2}{{N_z}^2}$	Normalised version of DN
4	Dependence Variance (DV)	$\sum_{i=1}^{N_g}\sum_{j=1}^{N_d}p(i,j)(j-\mu)^2$, $\mu=\sum_{i=1}^{N_g}\sum_{j=1}^{N_d}p(i,j)j$	Measure the variance in dependencies in the image
5	GL Non-Uniformity (GLN)	$\frac{\sum_{i=1}^{N_g}(\sum_{j=1}^{N_d}P(i,j))^2}{N_z}$	Measure of the similarity of GL intensity value, lower GLN correlates with greater similarity in intensity values
6	GL Variance (GLV)	$\sum_{i=1}^{N_g}\sum_{j=1}^{N_d}p(i,j)(i-\mu)^2$, $\mu=\sum_{i=1}^{N_g}\sum_{j=1}^{N_d}p(i,j)i$	Variance in grey level in the image
7	High GL Emphasis (HGLE)	$\frac{\sum_{i=1}^{N_g}\sum_{j=1}^{N_d}P(i,j)i^2}{N_z}$	Measures the distribution of high grey-level values, with a higher value indicating a greater concentration of high grey-level values in the image
8	Large Dependence Emphasis (LDE)	$\frac{\sum_{i=1}^{N_g}\sum_{j=1}^{N_d}P(i,j)j^2}{N_z}$	Measure of the distribution of large dependencies, with a greater value indicative of larger dependence and more homogeneous texture
9	Large Dependence High GL Emphasis (LDHGLE)	$\frac{\sum_{i=1}^{N_g}\sum_{j=1}^{N_d}P(i,j)i^2j^2}{N_z}$	Measure the joint distribution of large dependence with higher grey-level values
10	Large Dependence Low GL Emphasis (LDLGLE)	$\frac{\sum_{i=1}^{N_g}\sum_{j=1}^{N_d}\frac{P(i,j)j^2}{i^2}}{N_z}$	Measure the joint distribution of a large dependence with lower grey-level values
11	Low GL Emphasis (LGLE)	$\frac{\sum_{i=1}^{N_g}\sum_{j=1}^{N_d}\frac{P(i,j)}{i^2}}{N_z}$	Measures the distribution of low grey-level values, with a higher value indicating a greater concentration of low grey-level values in the image
12	Small Dependence Emphasis (SDE)	$\frac{\sum_{i=1}^{N_g}\sum_{j=1}^{N_d}\frac{P(i,j)}{i^2}}{N_z}$	Measure of the distribution of the small dependencies, a greater value indicates a smaller

			dependence and less homogeneous texture.
13	Small Dependence High GL Emphasis (SDHGLE)	$\frac{\sum_{i=1}^{N_g}\sum_{j=1}^{N_d}\frac{P(i,j)i^2}{j^2}}{N_z}$	Measures the joint distribution of small dependence with higher grey-level values.
14	Small Dependence Low GL Emphasis (SDLGLE)	$\frac{\sum_{i=1}^{N_g}\sum_{j=1}^{N_d}\frac{P(i,j)}{i^2 j^2}}{N_z}$	Measures the joint distribution of small dependence with lower grey-level values.

Table S8: List of Grey Level Dependence Matrix (GLDM) features

Results

Optimal number of GLs

N° GLs	ICC threshold					
	**0.90**	**0.89**	**0.88**	**0.87**	**0.86**	**0.85**
**8**	461	489	517	543	556	568
**16**	525	570	605	624	636	648
**32**	545	574	603	624	640	659
**64**	555	589	613	626	652	667
**128**	533	584	607	622	635	654
**256**	531	568	592	612	623	640
**512**	540	576	593	608	621	631

Table S9: Number of robust features *vs* number of GLs used to quantize the images. Features were evaluated for all the VOIs (6 NAWM tracts and NAWM), leading to a total number of 749 (= 7 VOIs * 107 features). A feature was considered robust when the ICC over the 4 QSM reconstructions was higher than a set threshold, from 0.85 to 0.90 (GL = Grey Level, VOI = Volume of Interest, NAWM = Normal Appearing White Matter, ICC = Intra-class Correlation Coefficient, QSM = Quantitative Susceptibility Mapping)

Robustness analysis

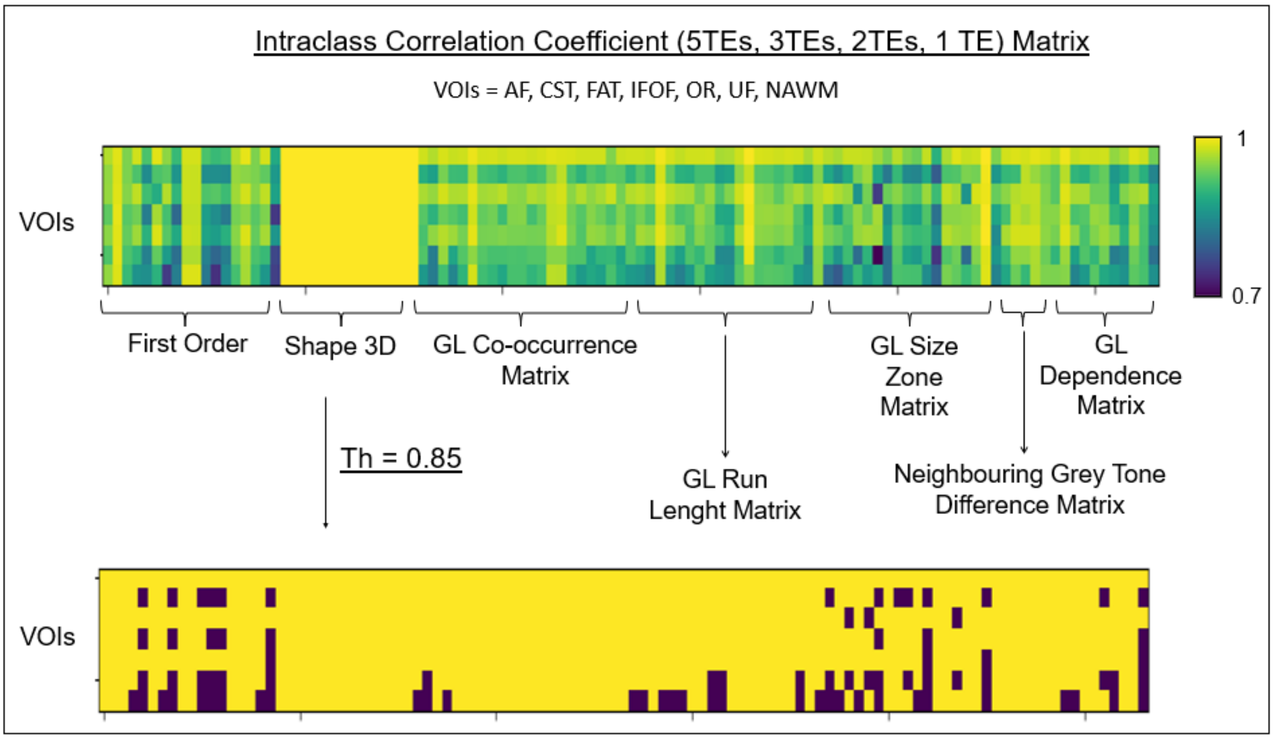

Figure S1: Robustness analysis: Intraclass correlation coefficient matrix
ICC matrix (n° GLs = 64) for the six NAWM tracts (AF, CST, FAT, IFOF, OR, UF) and for the entire NAWM (7 VOIs * 107 features = 749). In the bottom panel, the same matrix is shown after an upper-thresholding operation, setting the threshold as 0.85, leaving the robust features (ICC > 0.85) in yellow and the non-robust ones (ICC < 0.85) in purple (ICC = Intra-class Correlation Coefficient, GL = Grey Level, NAWM = Normal Appearing White Matter, AF = Arcuate Fasciculus, CST = Cortico-Spinal Tract, FAT = Frontal Aslant Tract, IFOF = Inferior Fronto-Occipital Fasciculus, OR = Optic Radiation, UF = Uncinate Fasciculus, VOI = Volume Of Interest)

	**NAWM Tracts**						**NAWM**
	**AF**	**CST**	**FAT**	**IFOF**	**OR**	**UF**	
**FO (18)**	18	12	18	13	17	12	9
**GLCM (24)**	24	24	24	24	24	23	19
**GLRLM (16)**	16	16	16	16	16	13	10
**GLSZM (16)**	16	11	13	14	15	9	9
**NGTDM (5)**	5	4	5	5	4	4	4
**GLDM (14)**	14	12	14	13	13	11	10
**TOT (93)**	93	79	90	85	89	72	61

Table S10: Number of robust features in NAWM Tracts and NAWM, divided into the different categories. Features were extracted setting 64 as binning and ICC was evaluated between the 4 QSM reconstructions, setting 0.85 as threshold (NAWM = Normal Appearing White Matter, ICC = Intra-class Correlation Coefficient, AF = Arcuate Fasciculus, CST = Cortico-Spinal Tract, FAT = Frontal Aslant Tract, IFOF = Inferior Fronto-Occipital Fasciculus, OR = Optic Radiation, UF = Uncinate Fasciculus, FO = First Order, GL = Grey Level, GLCM = GL Co-occurrence Matrix, GLRLM = GL Run Length Matrix, GLZM = GL Zone Matrix, NGTDM = Neighbouring Grey Tone Difference Matrix, GLDM = GL Dependence Matrix)

First-order statistics analysis

GLCM features	NAWM Tracts						NAWM
	AF	CST	FAT	IFOF	OR	UF	
Autocorrelation	0.977	0.877	0.958	0.914	0.905	0.910	0.849
Cluster Prominence	0.962	0.857	0.948	0.883	0.945	0.810	0.808
Cluster Shade	0.980	0.932	0.978	0.924	0.963	0.949	0.899
Cluster Tendency	0.972	0.896	0.960	0.913	0.952	0.865	0.821
Contrast	0.975	0.895	0.960	0.901	0.931	0.912	0.886
Correlation	0.990	0.955	0.987	0.976	0.986	0.944	0.978
Difference Average	0.978	0.902	0.955	0.922	0.932	0.912	0.898
Difference Entropy	0.979	0.897	0.958	0.930	0.933	0.904	0.888
Difference Variance	0.973	0.883	0.966	0.896	0.937	0.904	0.868
Id	0.978	0.907	0.943	0.939	0.934	0.906	0.904
Idm	0.978	0.908	0.942	0.940	0.934	0.906	0.904
Idmn	0.975	0.895	0.960	0.902	0.931	0.912	0.887
Idn	0.978	0.903	0.954	0.924	0.933	0.912	0.899
Imc1	0.984	0.939	0.971	0.969	0.965	0.941	0.966
Imc2	0.981	0.966	0.979	0.972	0.983	0.942	0.964
Inverse Variance	0.980	0.906	0.943	0.931	0.934	0.912	0.883
Joint Average	0.976	0.885	0.962	0.913	0.911	0.910	0.850
Joint Energy	0.977	0.910	0.946	0.940	0.947	0.865	0.869
Joint Entropy	0.977	0.900	0.957	0.928	0.944	0.889	0.859
MCC	0.938	0.918	0.973	0.908	0.928	0.892	0.904
Maximum Probability	0.973	0.916	0.931	0.938	0.949	0.862	0.870
Sum Average	0.976	0.885	0.962	0.913	0.911	0.910	0.850
Sum Entropy	0.977	0.911	0.969	0.932	0.956	0.892	0.842
Sum Squares	0.972	0.894	0.958	0.910	0.949	0.865	0.822

Table S11: ICC values (n° GLs = 64) for the GLCM features in the analysed NAWM VOIs. Considering 0.85 as the threshold, the robust features are highlighted in yellow, the others in purple. For features abbreviations, please refer to Tab. S4 (ICC = Intra-class Correlation Coefficient, GL = Grey Level, GLCM = Grey Level Co-occurrence Matrix, NAWM = Normal Appearing White Matter, VOI = Volume Of Interest, AF = Arcuate Fasciculus; CST = Cortico-Spinal Tract; FAT = Frontal Aslant Tract; IFOF = Inferior Fronto-Occipital Fasciculus; OR = Optic Radiation; UF = Uncinate Fasciculus)

GLRLM features	NAWM Tracts						NAWM
	AF	CST	FAT	IFOF	OR	UF	
GLN	0.993	0.979	0.989	0.977	0.987	0.960	0.959
GLNN	0.979	0.918	0.970	0.935	0.955	0.894	0.841
GLV	0.970	0.881	0.959	0.905	0.944	0.859	0.814
HGLRE	0.976	0.876	0.957	0.913	0.905	0.908	0.847
LRE	0.976	0.902	0.926	0.948	0.938	0.890	0.909
LRHGLRE	0.977	0.902	0.951	0.930	0.922	0.920	0.860
LRLGLE	0.957	0.856	0.908	0.870	0.904	0.828	0.846
LGLRE	0.959	0.864	0.937	0.867	0.881	0.825	0.837
RE	0.975	0.943	0.975	0.931	0.961	0.904	0.911
RLN	0.997	0.971	0.994	0.990	0.995	0.988	0.939

RLNN	0.980	0.914	0.946	0.950	0.943	0.909	0.909
RP	0.979	0.912	0.940	0.950	0.941	0.905	0.914
RV	0.975	0.899	0.918	0.946	0.935	0.886	0.911
SRE	0.979	0.912	0.944	0.950	0.943	0.904	0.906
SRHGLE	0.976	0.873	0.958	0.910	0.902	0.905	0.850
SRLGLH	0.959	0.863	0.938	0.866	0.876	0.823	0.837

Table S12: ICC values (n° GLs = 64) for the GLRLM features in the analysed NAWM VOIs. Considering 0.85 as the threshold, the robust features are highlighted in yellow, the others in purple. For features abbreviations, please refer to Tab. S5 (ICC = Intra-class Correlation Coefficient, GL = Grey Level, GLRLM = Grey Level Run Length Matrix, NAWM = Normal Appearing White Matter, VOI = Volume Of Interest, AF = Arcuate Fasciculus, CST = Cortico-Spinal Tract, FAT = Frontal Aslant Tract, IFOF = Inferior Fronto-Occipital Fasciculus, OR = Optic Radiation, UF = Uncinate Fasciculus)

GLSZM features	NAWM Tracts						NAWM
	AF	CST	FAT	IFOF	OR	UF	
GLN	0.977	0.938	0.960	0.958	0.960	0.946	0.944
GLNN	0.965	0.866	0.972	0.910	0.934	0.889	0.790
GLV	0.957	0.833	0.959	0.865	0.921	0.840	0.764
HGLZE	0.972	0.870	0.950	0.916	0.904	0.897	0.834
LAE	0.972	0.934	0.840	0.946	0.947	0.788	0.892
LAHGLE	0.969	0.952	0.931	0.921	0.956	0.885	0.847
LALGLE	0.953	0.866	0.730	0.956	0.952	0.670	0.873
LGLZE	0.932	0.803	0.925	0.837	0.857	0.790	0.793
SZN	0.965	0.891	0.941	0.909	0.928	0.913	0.907
SZNN	0.937	0.826	0.917	0.865	0.903	0.862	0.904
SAE	0.926	0.820	0.908	0.856	0.893	0.850	0.902
SAHGLE	0.969	0.866	0.951	0.910	0.898	0.891	0.832
SALGLE	0.881	0.768	0.896	0.794	0.830	0.720	0.776
ZE	0.959	0.907	0.970	0.943	0.964	0.910	0.934
ZP	0.984	0.910	0.956	0.943	0.953	0.927	0.921
ZV	0.973	0.935	0.840	0.947	0.947	0.786	0.892

Table S13: ICC values (n° GLs = 64) for the GLSZM features in the analysed NAWM VOIs. Considering 0.85 as the threshold, the robust features are highlighted in yellow, the others in purple. For features abbreviations, please refer to Tab. S6 (ICC = Intra-class Correlation Coefficient, GL = Grey Level, GLSZM = Grey Level Size Zone Matrix, NAWM = Normal Appearing White Matter, VOI = Volume Of Interest, AF = Arcuate Fasciculus, CST = Cortico-Spinal Tract, FAT = Frontal Aslant Tract, IFOF = Inferior Fronto-Occipital Fasciculus, OR = Optic Radiation, UF = Uncinate Fasciculus)

NGTDM features	NAWM Tracts						NAWM
	AF	CST	FAT	IFOF	OR	UF	
Busyness	0.976	0.930	0.981	0.952	0.960	0.939	0.918
Coarseness	0.998	0.993	0.990	0.996	0.998	0.983	0.984
Complexity	0.936	0.844	0.923	0.864	0.830	0.844	0.817
Contrast	0.978	0.890	0.942	0.904	0.958	0.895	0.867
Strength	0.983	0.894	0.963	0.969	0.978	0.947	0.926

Table S14: ICC values (n° GLs = 64) for the NGTDM features in the analysed NAWM VOIs. Considering 0.85 as the threshold, the robust features are highlighted in yellow, the others in purple. For features abbreviations, please refer to Tab. S7 (ICC = Intra-class Correlation Coefficient, GL = Grey Level, NGTDM = Neighbouring Grey Tone Difference Matrix, NAWM = Normal Appearing White Matter, VOI = Volume Of

Interest, AF = Arcuate Fasciculus; CST = Cortico-Spinal Tract; FAT = Frontal Aslant Tract; IFOF = Inferior Fronto-Occipital Fasciculus; OR = Optic Radiation; UF = Uncinate Fasciculus)

GLDM features	NAWM Tracts						NAWM
	AF	CST	FAT	IFOF	OR	UF	
DE	0.978	0.967	0.969	0.956	0.977	0.945	0.891
DN	0.986	0.949	0.971	0.970	0.979	0.959	0.958
DNN	0.982	0.931	0.937	0.942	0.944	0.926	0.937
DV	0.970	0.915	0.914	0.941	0.932	0.887	0.935
GLN	0.992	0.964	0.984	0.973	0.982	0.951	0.941
GLV	0.971	0.887	0.959	0.906	0.946	0.860	0.818
HGLE	0.976	0.878	0.958	0.913	0.905	0.909	0.848
LDE	0.975	0.907	0.927	0.949	0.937	0.888	0.914
LDHGLE	0.977	0.922	0.927	0.943	0.952	0.930	0.870
LDLGLE	0.947	0.846	0.864	0.887	0.935	0.811	0.855
LGLE	0.960	0.869	0.937	0.868	0.883	0.825	0.839
SDE	0.983	0.909	0.954	0.941	0.950	0.926	0.917
SDHGLE	0.973	0.879	0.961	0.907	0.905	0.901	0.904
SDLGLE	0.931	0.820	0.864	0.847	0.832	0.791	0.835

Table S15: ICC values (n° GLs = 64) for the GLDM features in the analysed NAWM VOIs. Considering 0.85 as the threshold, the robust features are highlighted in yellow, the others in purple. For features abbreviations, please refer to Tab. S8 (ICC = Intra-class Correlation Coefficient, GL = Grey Level, GLDM = GL Dependence Matrix, NAWM = Normal Appearing White Matter, VOI = Volume Of Interest, AF = Arcuate Fasciculus, CST = Cortico-Spinal Tract, FAT = Frontal Aslant Tract, IFOF = Inferior Fronto-Occipital Fasciculus, OR = Optic Radiation, UF = Uncinate Fasciculus)

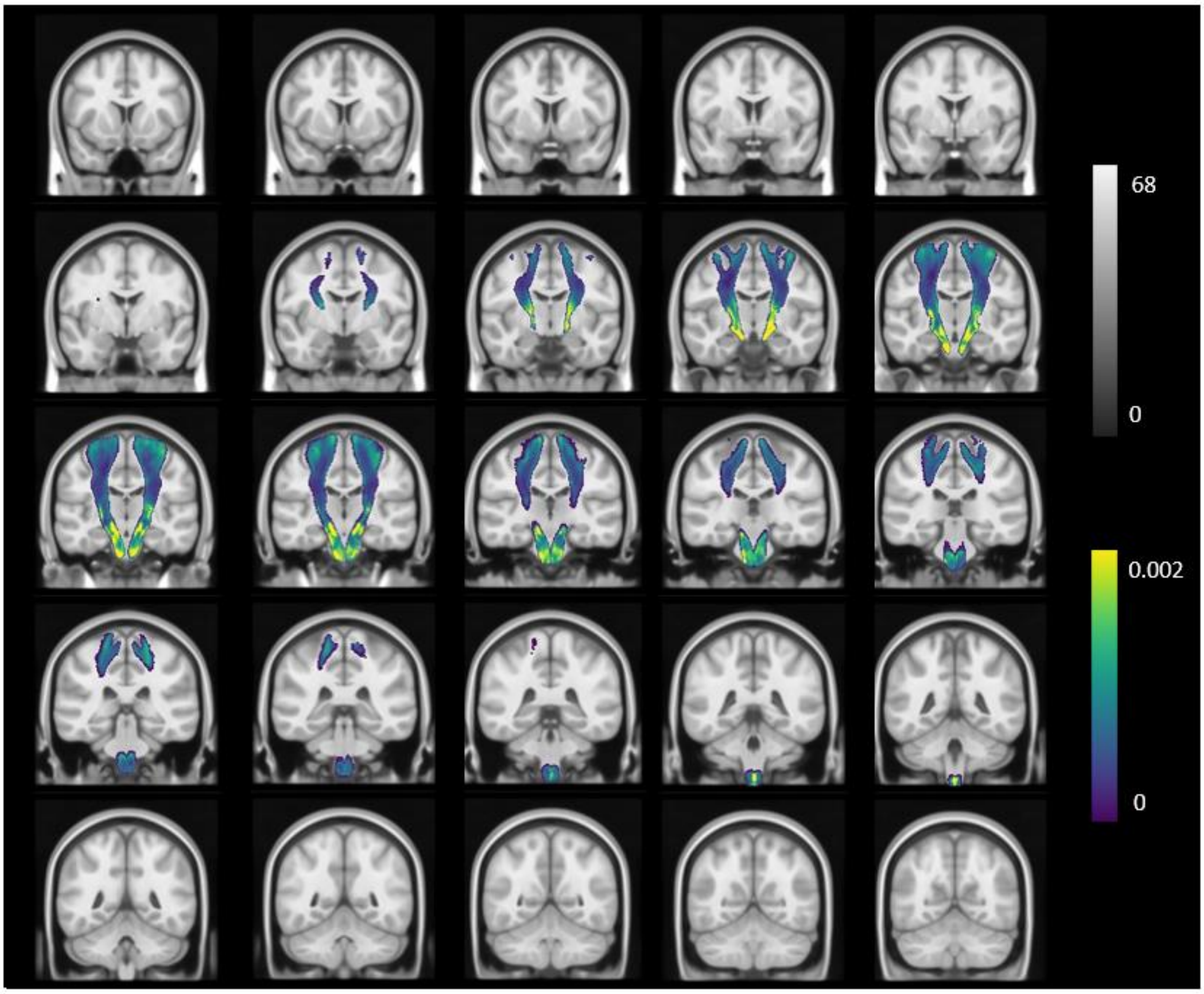

Figure S2: Variance of susceptibility in the CST in MNI152 space – coronal slices
QSM atlas was obtained non-linearly registering 30 healthy control exams to the MNI152 space, and a pixel-wise variance of the susceptibility measurements map was evaluated; in the figure, susceptibility variance of CST in MNI152 space (coronal slices) (QSM = Quantitative Susceptibility Mapping, CST = Cortico-Spinal Tract, MNI152 = Montreal Neurological Institute's 152 space)

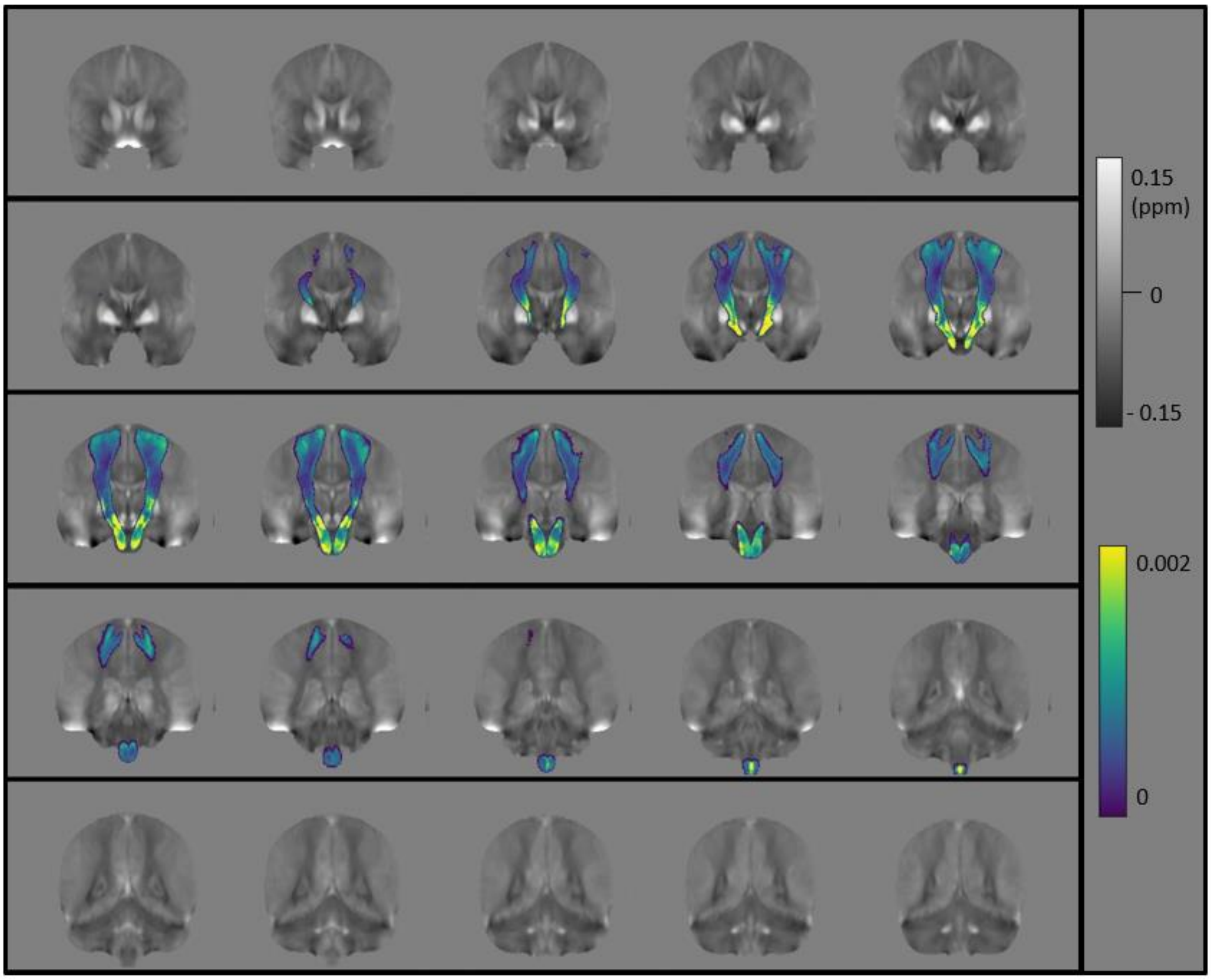

Figure S3: Variance of susceptibility in the CST overlaid on locally generated QSM atlas – coronal slices QSM atlas was obtained non-linearly registering 30 healthy control exams to the MNI152 space, and a pixel-wise variance of the susceptibility measurements map was evaluated; in the figure, susceptibility variance of CST in QSM atlas (coronal slices) (QSM = Quantitative Susceptibility Mapping, CST = Cortico-Spinal Tract, MNI152 = Montreal Neurological Institute's 152 space)

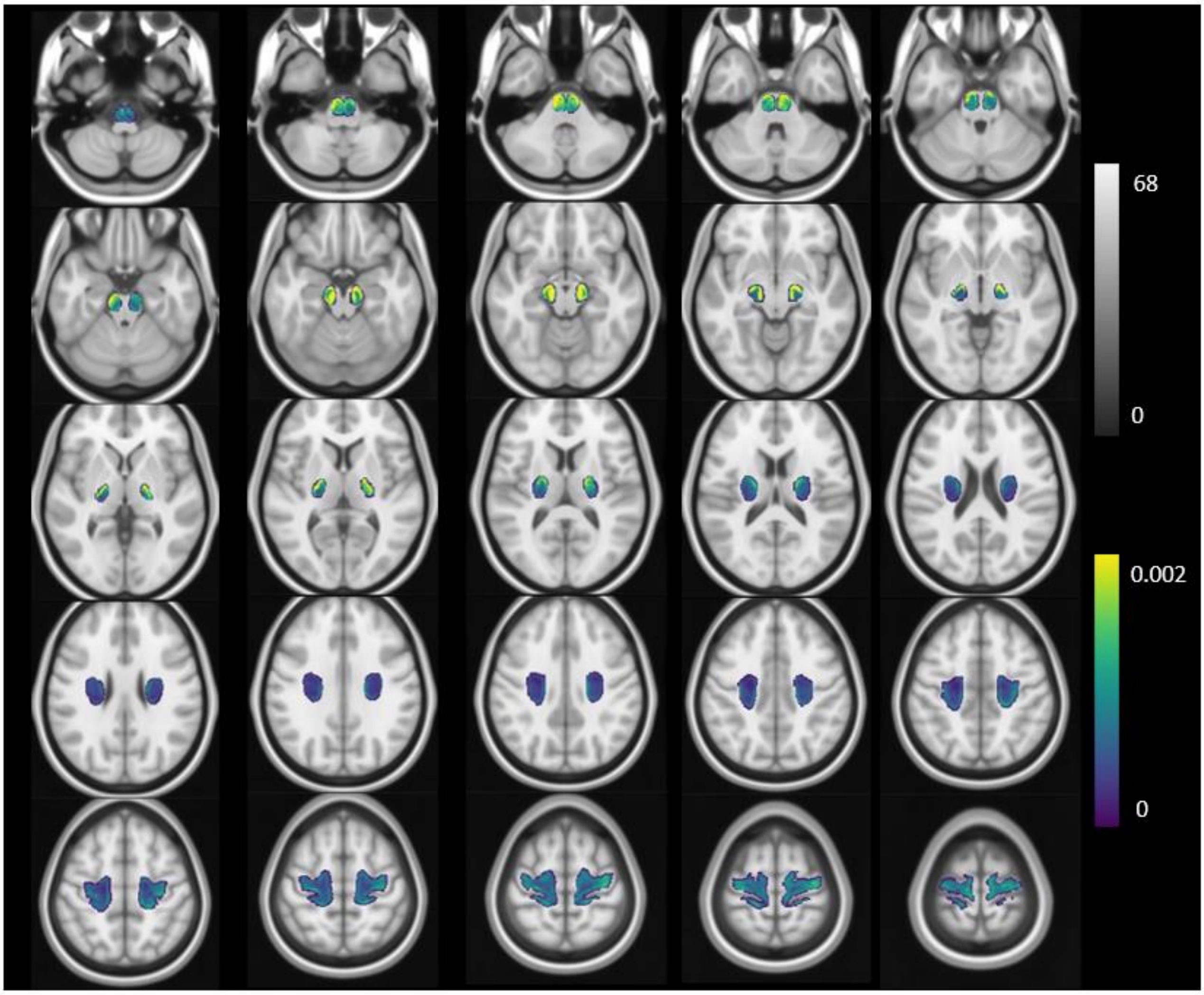

Figure S4: Variance of susceptibility in the CST in MNI152 space – axial slices
QSM atlas was obtained non-linearly registering 30 healthy control exams to the MNI152 space, and a pixel-wise variance of the susceptibility measurements map was evaluated; in the figure, susceptibility variance of CST in MNI152 (axial slices) (QSM = Quantitative Susceptibility Mapping, CST = Cortico-Spinal Tract, MNI152 = Montreal Neurological Institute's 152 space)

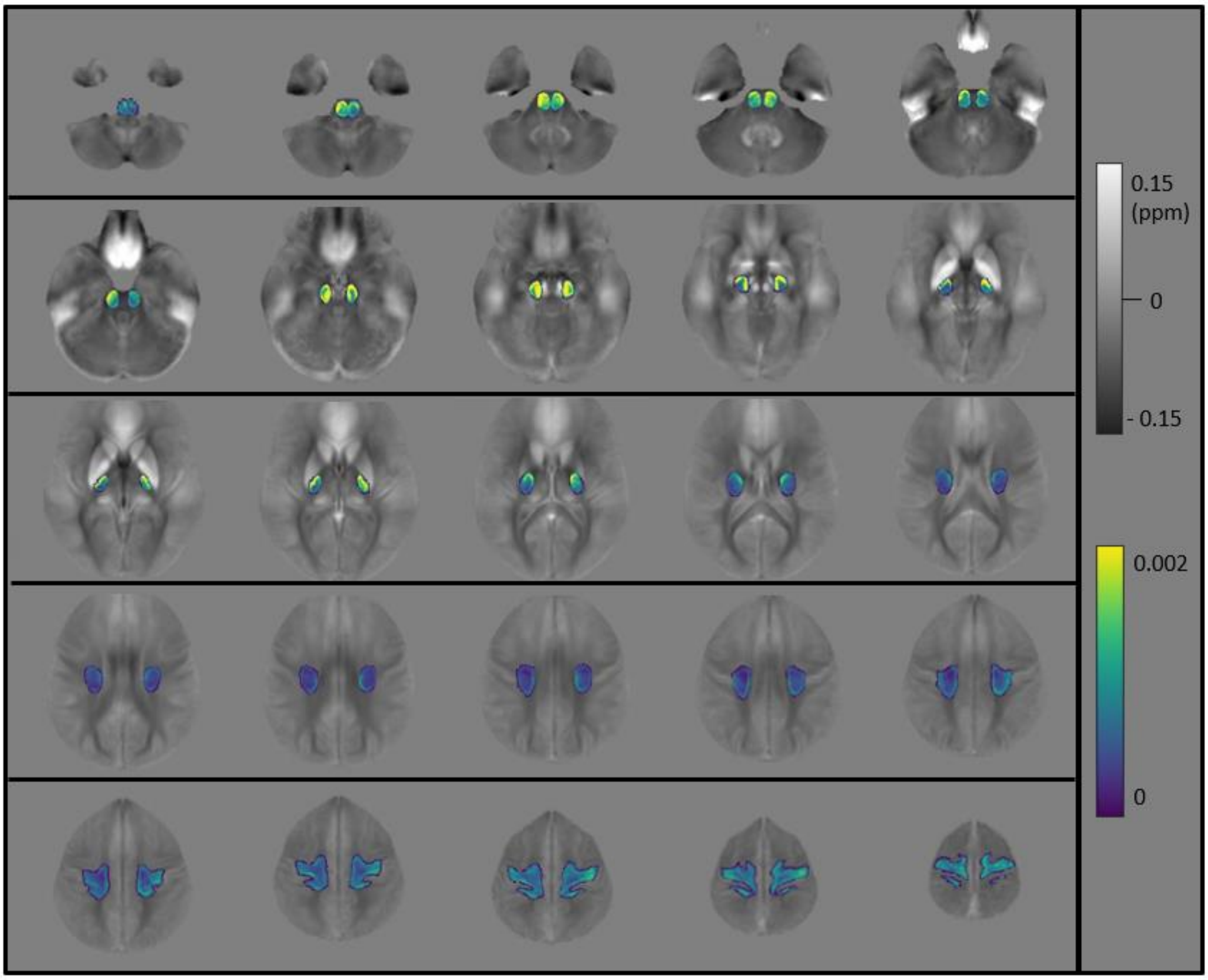

Figure S5: Variance of susceptibility in the CST overlaid on locally generated QSM atlas – axial slices
QSM atlas was obtained non-linearly registering 30 healthy control exams to the MNI152 space, and a pixel-wise variance of the susceptibility measurements map was evaluated; in the figure, susceptibility variance of CST in QSM atlas (axial slices) (QSM = Quantitative Susceptibility Mapping, CST = Cortico-Spinal Tract, MNI152 = Montreal Neurological Institute's 152 space)

Texture analysis

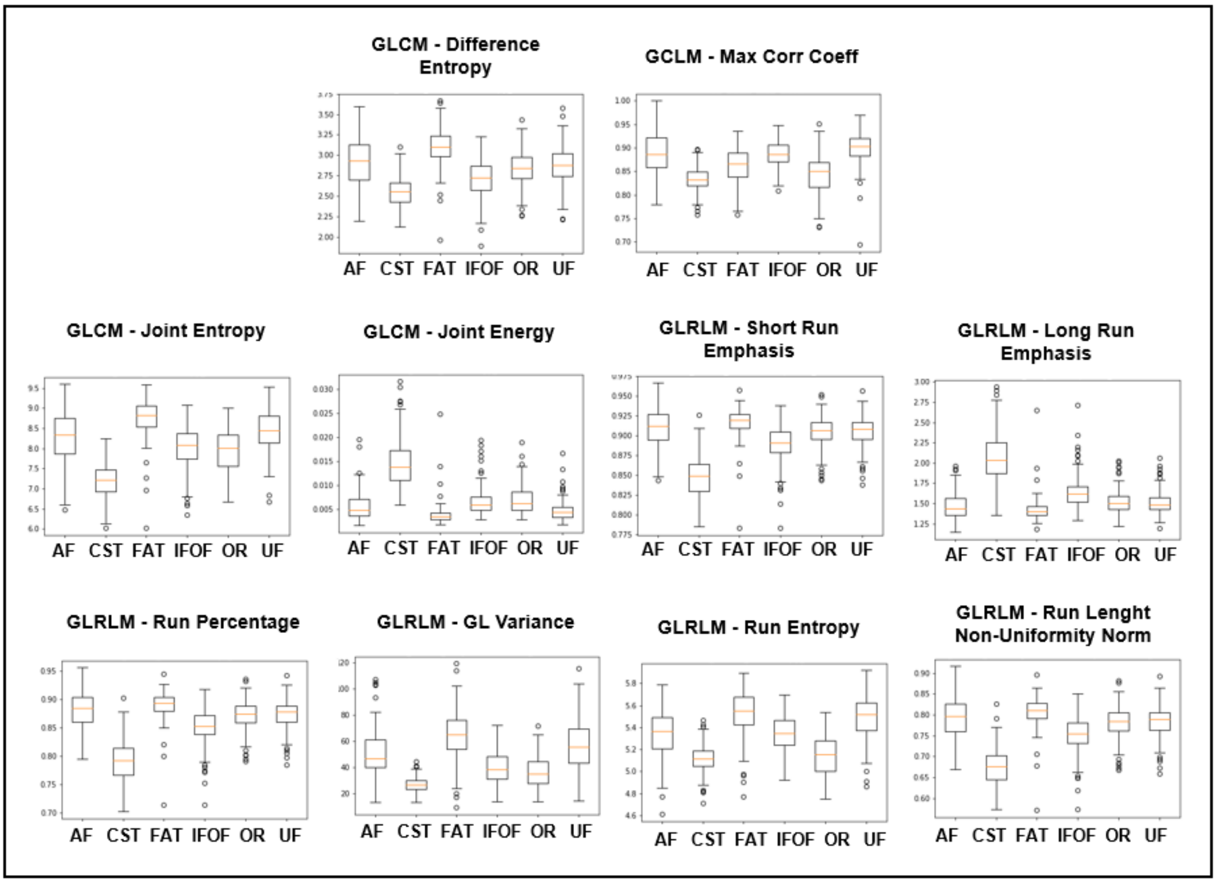

Figure S6: Texture features in NAWM tracts

Box plots showing texture features in NAWM tracts (5TE-QSM), in GLCM (difference entropy, maximal correlation coefficient, joint entropy, joint energy), and GLRLM (short run emphasis, run percentage, grey level variance, run entropy, run length non-uniformity normalised). CST is the one showing more uniformity in terms of texture, despite its wide extension (NAWM = Normal Appearing White Matter, TE = Time of Echo, QSM = Quantitative Susceptibility Mapping, GL = Grey Levels, GLCM = GL Co-occurrence Matrix, GLRLM = GL Run Length Matrix, AF = Arcuate Fasciculus, CST = Cortico-Spinal Tract, FAT = Frontal Aslant Tract, IFOF = Inferior Fronto-Occipital Fasciculus, OR = Optic Radiation, UF = Uncinate Fasciculus)

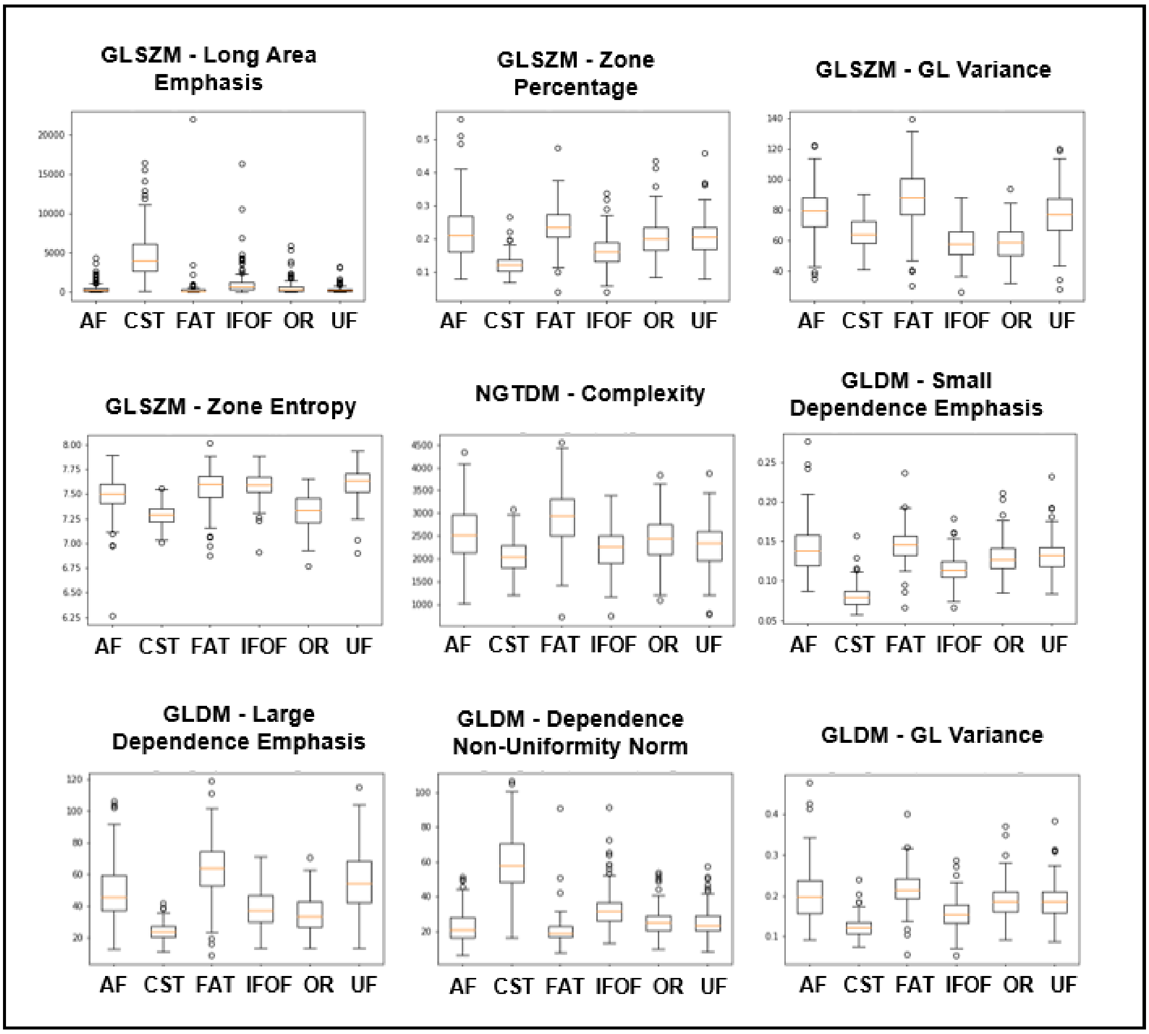

Figure S7: Texture features in NAWM tracts

Box plots showing texture features in NAWM tracts (5TE-QSM), in GLSZM (long area emphasis, zone percentage, grey level variance, zone entropy), NGTDM (complexity), and GLDM (small dependence emphasis, large dependence emphasis, dependence non-uniformity normalised, grey level variance). CST is the one showing more uniformity in terms of texture, despite its wide extension (NAWM = Normal Appearing White Matter, TE = Time of Echo, QSM = Quantitative Susceptibility Mapping, GL = Grey Levels, GLSZM = GL Size Zone Matrix, NGDTM = Neighbouring Grey Difference Tone Matrix, GLDM = GL Dependence Matrix, AF = Arcuate Fasciculus, CST = Cortico-Spinal Tract, FAT = Frontal Aslant Tract, IFOF = Inferior Fronto-Occipital Fasciculus, OR = Optic Radiation, UF = Uncinate Fasciculus)

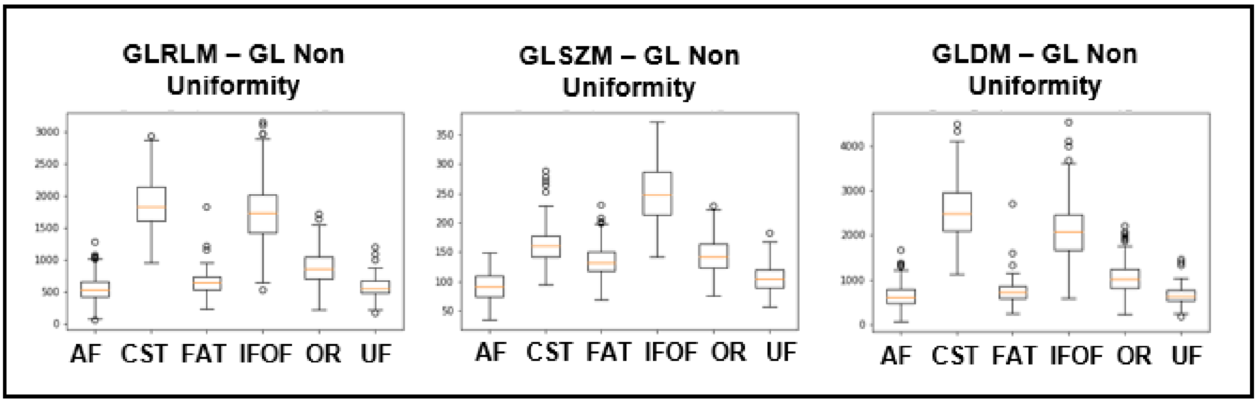

Figure S8: GL non-uniformity in NAWM tracts
Box plots showing GL Non Uniformity in NAWM tracts (5TE-QSM) , in GLRLM, GLSZM and GLDM, measure of variability in intensity values looking into run lengths, zones and dependences respectively; note the three measures are in agreement with each other, showing higher variability for CST and IFOF (NAWM = Normal Appearing White Matter, TE = Time of Echo, QSM = Quantitative Susceptibility Mapping, GL = Grey Levels, GLRLM = GL Run Lengths Matrix, GLSZM = GL Size Zone Matrix, GLDM = GL Dependence Matrix, AF = Arcuate Fasciculus, CST = Cortico-Spinal Tract, FAT = Frontal Aslant Tract, IFOF = Inferior Fronto-Occipital Fasciculus, OR = Optic Radiation, UF = Uncinate Fasciculus)

Robustness analysis in NAWM Tracts, L and R

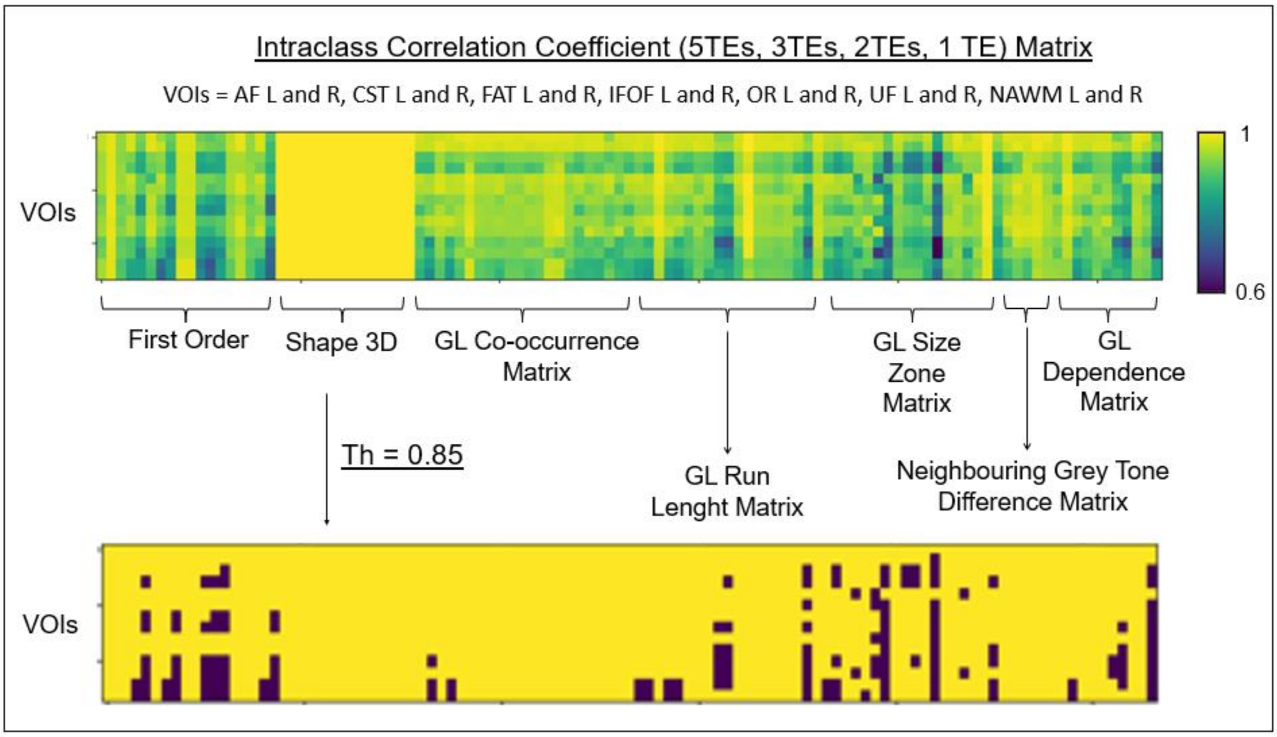

Figure S9: Robustness sub-analysis by side: Intraclass correlation coefficient matrix
ICC matrix (n° GLs = 64) for the six NAWM tracts (AF, CST, FAT, IFOF, OR, UF) and for the entire NAWM, dividing left and right hemispheres (2 sides * 7 VOIs * 107 features = 1498). In the bottom panel, the same matrix is shown after an upper-thresholding operation, setting the threshold as 0.85, leaving the robust features (ICC > 0.85) in yellow and the non-robust ones (ICC < 0.85) in purple (ICC = Intraclass Correlation Coefficient, GL = Grey Level, NAWM = Normal Appearing White Matter, AF = Arcuate Fasciculus, CST = Cortico-Spinal Tract, FAT = Frontal Aslant Tract, IFOF = Inferior Fronto-Occipital Fasciculus, OR = Optic Radiation, UF = Uncinate Fasciculus, VOI = Volume Of Interest)

Robust features across all VOIs

LEFT + RIGHT (ICC, nGLs = 64)		AF	CST	FAT	IFOF	OR	UF	NAWM
FO	10th Percentile	0.962	0.946	0.948	0.919	0.942	0.909	0.947
	90th Percentile	0.985	0.982	0.984	0.988	0.986	0.986	0.982
	Energy	0.936	0.935	0.94	0.915	0.922	0.906	0.892
	Kurtosis	0.986	0.907	0.874	0.956	0.982	0.921	0.881
	Mean	0.979	0.973	0.973	0.969	0.974	0.961	0.983
	Median	0.979	0.972	0.972	0.97	0.973	0.96	0.982
	RMS	0.964	0.947	0.953	0.93	0.942	0.925	0.903
	Skewness	0.988	0.943	0.978	0.943	0.981	0.958	0.956
	Total energy	0.936	0.935	0.94	0.915	0.922	0.906	0.892
GLCM	Cluster shade	0.98	0.932	0.978	0.924	0.963	0.949	0.899
	Contrast	0.975	0.895	0.96	0.901	0.931	0.912	0.886
	Correlation	0.99	0.955	0.987	0.976	0.986	0.944	0.978
	Difference average	0.978	0.902	0.955	0.922	0.932	0.912	0.898
	Difference entropy	0.979	0.897	0.958	0.93	0.933	0.904	0.888
	Difference variance	0.973	0.883	0.966	0.896	0.937	0.904	0.868
	Inverse difference	0.978	0.907	0.943	0.939	0.934	0.906	0.904
	Inverse difference moment	0.978	0.908	0.942	0.94	0.934	0.906	0.904
	Inverse difference moment norm	0.975	0.895	0.96	0.902	0.931	0.912	0.887
	Inverse difference norm	0.978	0.903	0.954	0.924	0.933	0.912	0.899
	Information measure of correlation 1	0.984	0.939	0.971	0.969	0.965	0.941	0.966
	Information measure of correlation 1	0.981	0.966	0.979	0.972	0.983	0.942	0.964
	Inverse variance	0.98	0.906	0.943	0.931	0.934	0.912	0.883
	Joint average	0.976	0.885	0.962	0.913	0.911	0.91	0.85
	Joint energy	0.977	0.91	0.946	0.94	0.947	0.865	0.869
	Joint entropy	0.977	0.9	0.957	0.928	0.944	0.889	0.859
	Maximal correlation coefficient	0.938	0.918	0.973	0.908	0.928	0.892	0.904
	Maximum probability	0.973	0.916	0.931	0.938	0.949	0.862	0.87
	Sum average	0.976	0.885	0.962	0.913	0.911	0.91	0.85
GLRLM (R = Run)	GL non uniformity	0.993	0.979	0.989	0.977	0.987	0.96	0.959
	Long R emphasis	0.976	0.902	0.926	0.948	0.938	0.89	0.909
	Long R high GL emphasis	0.977	0.902	0.951	0.93	0.922	0.92	0.86
	R Entropy	0.975	0.943	0.975	0.931	0.961	0.904	0.911
	R length non uniformity	0.997	0.971	0.994	0.99	0.995	0.988	0.939
	R length non uniformity norm	0.98	0.914	0.946	0.95	0.943	0.909	0.909
	R percentage	0.979	0.912	0.94	0.95	0.941	0.905	0.914
	R variance	0.975	0.899	0.918	0.946	0.935	0.886	0.911
	Short R emphasis	0.979	0.912	0.944	0.95	0.943	0.904	0.906
	Short R high GL emphasis	0.976	0.873	0.958	0.91	0.902	0.905	0.85
GLSZM (S = Size)	GL non uniformity	0.977	0.938	0.96	0.958	0.96	0.946	0.944
	Size Z non uniformity	0.965	0.891	0.941	0.909	0.928	0.913	0.907
	Z entropy	0.959	0.907	0.97	0.943	0.964	0.91	0.934
	Z percentage	0.984	0.91	0.956	0.943	0.953	0.927	0.921
NGTDM	Busyness	0.976	0.93	0.981	0.952	0.96	0.939	0.918

	Coarseness	0.998	0.993	0.99	0.996	0.998	0.983	0.984
	Contrast	0.978	0.89	0.942	0.904	0.958	0.895	0.867
	Strength	0.983	0.894	0.963	0.969	0.978	0.947	0.926
**GLDM (D = Dependence)**	D entropy	0.978	0.967	0.969	0.956	0.977	0.945	0.891
	D non uniformity	0.986	0.949	0.971	0.97	0.979	0.959	0.958
	D non uniformity norm	0.982	0.931	0.937	0.942	0.944	0.926	0.937
	D variance	0.97	0.915	0.914	0.941	0.932	0.887	0.935
	GL non uniformity	0.992	0.964	0.984	0.973	0.982	0.951	0.941
	Large D emphasis	0.975	0.907	0.927	0.949	0.937	0.888	0.914
	Large D high GL emphasis	0.977	0.922	0.927	0.943	0.952	0.93	0.87
	Small D emphasis	0.983	0.909	0.954	0.941	0.95	0.926	0.917
	Small D high GL emphasis	0.973	0.879	0.961	0.907	0.905	0.901	0.904

Table S16: List of robust features (ICC > 0.85) with the corresponding ICC value in the analysed NAWM tracts and NAWM, considering left and right sides together (GL = Grey Level, GLDM = GL Dependence Matrix, NAWM = Normal Appearing White Matter, AF = Arcuate Fasciculus, CST = Cortico-Spinal Tract, FAT = Frontal Aslant Tract, IFOF = Inferior Fronto-Occipital Fasciculus, OR = Optic Radiation, UF = Uncinate Fasciculus)

**LEFT (ICC, nGLs = 64)**		**AF**	**CST**	**FAT**	**IFOF**	**OR**	**UF**	**NAWM**
**FO**	10th Percentile	0.962	0.944	0.948	0.914	0.942	0.905	0.946
	90th Percentile	0.984	0.981	0.983	0.987	0.984	0.986	0.982
	Energy	0.938	0.931	0.940	0.908	0.917	0.906	0.893
	Kurtosis	0.982	0.925	0.873	0.963	0.971	0.943	0.892
	Mean	0.980	0.972	0.972	0.967	0.972	0.959	0.983
	Median	0.980	0.970	0.970	0.969	0.970	0.959	0.982
	RMS	0.963	0.948	0.953	0.926	0.943	0.924	0.904
	Skewness	0.987	0.951	0.976	0.947	0.978	0.949	0.957
	Total energy	0.938	0.931	0.940	0.908	0.917	0.906	0.893
**GLCM**	Cluster shade	0.971	0.945	0.977	0.925	0.968	0.934	0.900
	Contrast	0.985	0.926	0.964	0.932	0.952	0.913	0.900
	Correlation	0.989	0.941	0.988	0.973	0.984	0.963	0.979
	Difference average	0.983	0.926	0.960	0.940	0.948	0.917	0.905
	Difference entropy	0.981	0.918	0.962	0.943	0.943	0.914	0.891
	Difference variance	0.982	0.918	0.966	0.929	0.951	0.908	0.881
	Inverse difference	0.980	0.924	0.951	0.948	0.941	0.922	0.906
	Inverse difference moment	0.980	0.925	0.950	0.948	0.940	0.922	0.906
	Inverse difference moment norm	0.985	0.925	0.965	0.932	0.952	0.913	0.900
	Inverse difference norm	0.983	0.926	0.959	0.941	0.947	0.918	0.905
	Information measure of correlation 1	0.985	0.937	0.973	0.967	0.972	0.960	0.966
	Information measure of correlation 1	0.981	0.948	0.980	0.972	0.981	0.951	0.965
	Inverse variance	0.982	0.930	0.951	0.944	0.942	0.921	0.904
	Joint average	0.973	0.899	0.965	0.927	0.938	0.888	0.867
	Joint energy	0.978	0.919	0.953	0.947	0.941	0.902	0.863
	Joint entropy	0.977	0.919	0.958	0.942	0.945	0.892	0.862
	Maximal correlation coefficient	0.933	0.895	0.972	0.924	0.909	0.923	0.893
	Maximum probability	0.973	0.914	0.935	0.941	0.938	0.873	0.864

	Sum average	0.973	0.899	0.965	0.927	0.938	0.888	0.867
**GLRLM (R = Run)**	GL non uniformity	0.993	0.981	0.992	0.981	0.987	0.967	0.953
	Long R emphasis	0.976	0.913	0.935	0.953	0.939	0.922	0.908
	Long R high GL emphasis	0.972	0.907	0.956	0.942	0.951	0.907	0.889
	R Entropy	0.974	0.940	0.975	0.945	0.961	0.905	0.884
	R length non uniformity	0.997	0.979	0.996	0.992	0.996	0.992	0.932
	R length non uniformity norm	0.980	0.929	0.953	0.955	0.946	0.928	0.908
	R percentage	0.979	0.925	0.949	0.955	0.943	0.927	0.913
	R variance	0.975	0.909	0.927	0.952	0.936	0.921	0.912
	Short R emphasis	0.979	0.926	0.951	0.955	0.945	0.927	0.904
	Short R high GL emphasis	0.973	0.888	0.964	0.926	0.938	0.890	0.876
**GLSZM (S = Size)**	GL non uniformity	0.982	0.951	0.969	0.956	0.964	0.962	0.933
	Size Z non uniformity	0.969	0.919	0.942	0.923	0.927	0.900	0.902
	Z entropy	0.958	0.871	0.968	0.936	0.956	0.911	0.934
	Z percentage	0.986	0.931	0.961	0.953	0.956	0.928	0.930
**NGTDM**	Busyness	0.977	0.940	0.985	0.951	0.950	0.922	0.919
	Coarseness	0.998	0.993	0.993	0.996	0.998	0.989	0.982
	Contrast	0.983	0.922	0.948	0.930	0.972	0.899	0.877
	Strength	0.978	0.906	0.969	0.963	0.976	0.966	0.914
**GLDM (D = Dependence)**	D entropy	0.980	0.961	0.971	0.961	0.973	0.955	0.882
	D non uniformity	0.987	0.960	0.976	0.973	0.980	0.963	0.948
	D non uniformity norm	0.987	0.946	0.948	0.952	0.956	0.933	0.940
	D variance	0.967	0.923	0.921	0.948	0.933	0.922	0.935
	GL non uniformity	0.991	0.970	0.988	0.978	0.983	0.961	0.936
	Large D emphasis	0.973	0.918	0.935	0.954	0.938	0.923	0.912
	Large D high GL emphasis	0.974	0.921	0.933	0.950	0.970	0.933	0.893
	Small D emphasis	0.986	0.930	0.960	0.952	0.955	0.929	0.923
	Small D high GL emphasis	0.980	0.891	0.966	0.909	0.938	0.892	0.920

Table S17: List of robust features (ICC > 0.85) with the corresponding ICC values in the analysed NAWM tracts and NAWM, considering the left side (GL = Grey Level, GLDM = GL Dependence Matrix, NAWM = Normal Appearing White Matter, AF = Arcuate Fasciculus, CST = Cortico-Spinal Tract, FAT = Frontal Aslant Tract, IFOF = Inferior Fronto-Occipital Fasciculus, OR = Optic Radiation, UF = Uncinate Fasciculus)

**RIGHT (ICC, nGLs = 64)**		**AF**	**CST**	**FAT**	**IFOF**	**OR**	**UF**	**NAWM**
**FO**	10th Percentile	0.963	0.948	0.950	0.926	0.947	0.917	0.948
	90th Percentile	0.984	0.982	0.984	0.989	0.987	0.983	0.983
	Energy	0.938	0.941	0.946	0.923	0.931	0.911	0.894
	Kurtosis	0.984	0.911	0.948	0.957	0.977	0.911	0.883
	Mean	0.978	0.974	0.974	0.971	0.976	0.963	0.983
	Median	0.978	0.973	0.972	0.971	0.975	0.962	0.982
	RMS	0.965	0.948	0.954	0.936	0.945	0.930	0.904
	Skewness	0.986	0.946	0.978	0.957	0.980	0.955	0.958
	Total energy	0.938	0.941	0.946	0.923	0.931	0.911	0.894
**GLCM**	Cluster shade	0.985	0.941	0.978	0.955	0.957	0.930	0.897
	Contrast	0.970	0.885	0.962	0.947	0.940	0.938	0.889

	Correlation	0.982	0.961	0.979	0.976	0.986	0.929	0.977
	Difference average	0.976	0.894	0.955	0.946	0.941	0.934	0.904
	Difference entropy	0.979	0.893	0.955	0.940	0.941	0.925	0.894
	Difference variance	0.970	0.877	0.967	0.938	0.944	0.924	0.866
	Inverse difference	0.979	0.902	0.937	0.941	0.944	0.924	0.911
	Inverse difference moment	0.980	0.903	0.933	0.940	0.945	0.922	0.911
	Inverse difference moment norm	0.971	0.885	0.962	0.948	0.940	0.938	0.890
	Inverse difference norm	0.977	0.894	0.953	0.946	0.941	0.933	0.905
	Information measure of correlation 1	0.977	0.947	0.969	0.973	0.972	0.926	0.970
	Information measure of correlation 1	0.974	0.966	0.971	0.968	0.983	0.904	0.962
	Inverse variance	0.980	0.893	0.937	0.943	0.942	0.925	0.881
	Joint average	0.972	0.875	0.954	0.878	0.897	0.904	0.865
	Joint energy	0.978	0.910	0.925	0.921	0.970	0.895	0.868
	Joint entropy	0.979	0.893	0.945	0.926	0.949	0.906	0.862
	Maximal correlation coefficient	0.962	0.919	0.963	0.900	0.940	0.864	0.914
	Maximum probability	0.968	0.910	0.900	0.912	0.968	0.876	0.873
	Sum average	0.972	0.875	0.954	0.878	0.897	0.904	0.865
**GLRLM (R = Run)**	GL non uniformity	0.994	0.976	0.989	0.973	0.987	0.966	0.951
	Long R emphasis	0.979	0.901	0.918	0.935	0.948	0.916	0.917
	Long R high GL emphasis	0.972	0.890	0.952	0.890	0.901	0.908	0.853
	R Entropy	0.973	0.936	0.964	0.931	0.961	0.892	0.887
	R length non uniformity	0.999	0.970	0.997	0.994	0.996	0.993	0.947
	R length non uniformity norm	0.980	0.911	0.934	0.946	0.951	0.925	0.914
	R percentage	0.980	0.909	0.927	0.943	0.950	0.923	0.921
	R variance	0.978	0.898	0.909	0.929	0.947	0.913	0.919
	Short R emphasis	0.980	0.910	0.931	0.944	0.951	0.922	0.912
	Short R high GL emphasis	0.967	0.864	0.951	0.880	0.898	0.900	0.870
**GLSZM (S = Size)**	GL non uniformity	0.979	0.938	0.968	0.966	0.970	0.946	0.945
	Size Z non uniformity	0.961	0.875	0.948	0.933	0.928	0.913	0.914
	Z entropy	0.956	0.884	0.953	0.930	0.957	0.898	0.916
	Z percentage	0.982	0.894	0.950	0.961	0.955	0.941	0.928
**NGTDM**	Busyness	0.972	0.931	0.975	0.941	0.947	0.946	0.936
	Coarseness	0.997	0.992	0.993	0.995	0.997	0.980	0.983
	Contrast	0.970	0.865	0.951	0.945	0.950	0.927	0.868
	Strength	0.985	0.904	0.982	0.970	0.983	0.933	0.912
**GLDM (D = Dependence)**	D entropy	0.975	0.969	0.958	0.955	0.979	0.927	0.883
	D non uniformity	0.990	0.938	0.981	0.974	0.982	0.961	0.959
	D non uniformity norm	0.979	0.918	0.940	0.960	0.953	0.941	0.943
	D variance	0.970	0.911	0.883	0.920	0.948	0.899	0.942
	GL non uniformity	0.993	0.964	0.987	0.968	0.983	0.961	0.936
	Large D emphasis	0.978	0.906	0.910	0.933	0.949	0.911	0.922
	Large D high GL emphasis	0.977	0.912	0.941	0.917	0.921	0.925	0.865
	Small D emphasis	0.980	0.893	0.947	0.961	0.954	0.942	0.924
	Small D high GL emphasis	0.957	0.865	0.946	0.925	0.915	0.924	0.909

Table S18: List of robust features (ICC > 0.85) with the corresponding ICC value in the analysed NAWM tracts and NAWM, considering the right side (GL = Grey Level, GLDM = GL Dependence Matrix, NAWM = Normal Appearing White Matter, AF = Arcuate Fasciculus, CST = Cortico-Spinal Tract, FAT = Frontal Aslant Tract, IFOF = Inferior Fronto-Occipital Fasciculus, OR = Optic Radiation, UF = Uncinate Fasciculus)
